# Role of Bruton’s Tyrosine Kinase in mast cell driven urothelial barrier injury in an LL-37 induced model of interstitial cystitis

**DOI:** 10.1038/s41598-026-50443-z

**Published:** 2026-04-30

**Authors:** Guang Wang, Bin-sen Li, Jin-yi Chu, Xian Chen, Tong-xin Yang, Ke-wei Fang, Jiong-ming Li

**Affiliations:** The Second Affiliated Hospital of Kunming Medical University, Kunming, Yunnan, 650101 People’s Republic of China

**Keywords:** BTK, Interstitial cystitis, bladder pain syndrome, Mast cell, Bladder barrier, Cell biology, Urology

## Abstract

Interstitial cystitis/bladder pain syndrome (IC/BPS) is a chronic urinary disorder with unclear pathogenesis. Previous studies identified BTK as a hub gene potentially involved in IC/BPS. This study investigates BTK’s role in mast cell (MC) activation and bladder inflammation. An LL-37-induced IC/BPS rat model and an in vitro MCs Co-culture system were established. BTK expression was modulated via adenoviral vectors and cell transfection. Bladder inflammation, MC degranulation, and urothelial barrier function were assessed using histology, ELISA, RT-qPCR, Western blot, IHC, TEM, and IF. MC function was evaluated via CCK-8, flow cytometry, Transwell, and TEM. LL-37 upregulated BTK in IC/BPS rats, promoting inflammation, cytokine release, collagen deposition, MC degranulation, and urothelial damage. BTK overexpression exacerbated, while knockdown alleviated these effects. In vitro, LL-37 stimulated MC proliferation, invasion, and degranulation, and reduced apoptosis. Co-culture with activated MCs decreased glycosaminoglycan (GAG) and tight junction (TJ) proteins in SV-HUC-1 cells, enhanced by BTK overexpression and reversed by knockdown. BTK promotes LL-37-induced MC activation and urothelial barrier disruption by suppressing GAGs and TJ proteins, contributing to IC/BPS pathophysiology.

**Supplementary Information:**

The online version contains supplementary material available at 10.1038/s41598-026-50443-z.

## Introduction

Interstitial cystitis/bladder pain syndrome (IC/BPS) is a chronic bladder disorder with a rising incidence, yet its exact pathogenesis remains unclear. Due to the absence of well-defined etiological mechanisms and diagnostic criteria, IC/BPS is typically diagnosed by exclusion. However, the significant heterogeneity in clinical symptoms and pathophysiological features among patients poses major challenges for accurate diagnosis, effective disease management, and the development of targeted therapies.Mast cells (MCs) are immune cells of hematopoietic origin, derived from the bone marrow and yolk sac, and are widely distributed throughout loose connective tissues in the body. Previous studies have demonstrated that increased proliferation of mast cells (MCs) in bladder tissue may be associated with symptom onset in IC/BPS^1^. External inflammatory stimuli can activate MCs and trigger degranulation, releasing histamine, cytokines, and proteases that further promote inflammatory cell infiltration into the tissue^2^. This altered microenvironment reinforces MC activation and degranulation, forming a positive feedback loop that exacerbates IC/BPS symptoms.

Aberrant activation and degranulation of mast cells (MCs) are key contributors to the pathogenesis of various diseases. MC stabilizers alleviate disease symptoms by reducing MC activation and degranulation, thereby limiting the release of inflammatory mediators, histamine, and proteases. In IC/BPS, MC activation and degranulation have also been identified as pathological factors involved in disease development, a finding supported by clinical and pathological examinations^3,4^. However, the molecular mechanisms by which MC activation contributes to IC/BPS progression remain poorly understood. Therefore, elucidating the mechanisms that regulate MC proliferation and activation is crucial for developing strategies to suppress MC-mediated degranulation and inflammation in IC/BPS.

Bruton’s tyrosine kinase (BTK), a member of the TEC family of non-receptor tyrosine kinases, functions as a key intracellular signaling molecule predominantly expressed in B cells and various hematopoietic lineages. BTK is an essential component of the downstream signaling cascade of the B cell receptor (BCR), regulating B cell maturation, survival, activation, cytokine production, and antigen-dependent stimulation. In addition, BTK also plays roles in modulating the biological behavior of other immune and neuronally-derived cells^5,6^. Emerging evidence has shown that BTK can activate mast cells, promoting the production of eicosanoids and reactive oxygen species (ROS), thereby contributing to allergic responses^7^. However, whether BTK is involved in the pathogenesis and progression of IC/BPS has not yet been reported.

Intravesical LL-37 has been widely used to induce an IC/BPS-like cystitis. LL-37 can trigger urothelial injury/apoptosis and promote the release of danger signals such as ATP, thereby activating downstream inflammatory pathways and pain-related signaling. In addition, LL-37 has been reported to recruit inflammatory cells and promote mast cell activation/degranulation, contributing to sustained bladder inflammation and barrier dysfunction^2,8,9^.

Based on the above findings, we hypothesized that BTK may affect urothelial barrier function by modulating mast cell activation. Therefore, we established an LL-37-induced IC/BPS animal model to investigate the role of BTK expression in bladder inflammation, mast cell activation, urothelial barrier integrity, and its potential downstream signaling pathways. Activated MCs were also co-cultured with immortalized human ureteral epithelial cells (SV-HUC-1) to explore the impact of BTK on MC activation and the subsequent effects of MC activation on urothelial barrier function.

## Materials and methods

### Experimental design

BTK is expressed in a variety of immune cells, including B cells, macrophages, mast cells, basophils, and platelets, and participates in the regulation of both innate and adaptive immune responses. Based on our previous findings indicating that BTK regulates mast cell proliferation, invasion, and degranulation, an LL-37-induced IC/BPS rat model was established to investigate the functional role of BTK in vivo and to explore its underlying molecular mechanisms.

### Animal model and experimental design

To investigate the role of BTK in IC/BPS, an adeno-associated virus (AAV)-mediated gene overexpression or knockdown approach was employed in combination with intravesical instillation of LL-37 to induce an IC/BPS model. AAV vectors (1 × 10^11 vg/mL) were administered via tail-vein injection at a volume of 50 µL per rat. BTK modulation efficiency in bladder tissues was assessed by RT-qPCR (BTK mRNA) and Western blotting (BTK protein and downstream targets (Bcl-2, P21, and c-Myc)). Tissue localization was evaluated by immunohistochemistry for BTK- and tryptase-positive cells and by immunofluorescence staining observed under a fluorescence microscope. A total of 36 male.

Sprague–Dawley (SD) rats (6–8 weeks old, 200 ± 20 g) were randomly assigned into six groups (*n* = 6 per group): **sham-operated** group, **interstitial cystitis/bladder pain syndrome (IC/BPS)** group, IC/BPS + **overexpression negative control (OV-NC)** group, IC/BPS + **BTK overexpression (OV-BTK)** group, IC/BPS + **knockdown negative control (KD-NC)** group, and IC/BPS + **BTK knockdown (KD-BTK)** group.(Table 1).

On day 0, rats in the OV/KD groups received a single tail-vein injection of AAV (1 × 10¹¹ vg/mL, 50 µL/rat) carrying OV-NC, OV-BTK, KD-NC, or KD-BTK constructs; sham and IC/BPS groups received 50 µL sterile saline via tail vein. After 7 days (to allow viral expression), intravesical instillation was initiated to induce the IC/BPS model: 320 µM LL-37 (150 µL) was instilled into the bladder and retained for 1 h in the IC/BPS, IC/BPS + OV-NC, IC/BPS + OV-BTK, IC/BPS + KD-NC, and IC/BPS + KD-BTK groups, whereas the sham group received 150 µL sterile saline using the same procedure. LL-37 (or saline for sham) instillation was repeated on days 4 and 7 after the first instillation (i.e., three total instillations per rat).

**Table 1:** Grouping of animal models.

Groups	LL-37 Process	AAV Process
sham	None(normal saline)	None(normal saline)
IC/BPS	Yes	None(normal saline)
IC/BPS + OV-NC	Yes	negative control
IC/BPS + OV-BTK	Yes	BTK Overexpression
IC/BPS + KD-NC	Yes	negative control
IC/BPS + KD-BTK	Yes	BTK knockdown

^1^ The injection volume of normal saline in sham group and IC/BPS group was the same as that of AAV in other groups.

Pain assessment and tissue collection. On day 8 after the first intravesical instillation, pain-related responses were evaluated using Von Frey filaments (0.07 g, 0.4 g, 1.0 g, and 4.0 g). Scores were recorded as: 0 = no response; 1 = licking and/or body twitching; 2 = jumping. Immediately after behavioral testing, rats were euthanized and bladder tissues were harvested for hematoxylin and eosin (H&E) staining, Masson’s trichrome staining, immunohistochemistry (IHC), immunofluorescence (IF), enzyme-linked immunosorbent assay (ELISA), real-time quantitative PCR (RT-qPCR), Western blotting, and transmission electron microscopy (TEM).

### Animal anesthesia and euthanasia procedures

Adult male Sprague-Dawley rats (200 ± 20 g) were anesthetized by intraperitoneal injection of sodium pentobarbital (50 mg/kg), diluted 1:3 in sterile saline. Adequate anesthesia was confirmed by the absence of pedal-withdrawal and palpebral reflexes, and body temperature was maintained at 37 °C on a thermostatic pad throughout the procedure. At the experimental endpoint (day 8 after the first intravesical instillation), immediately after Von Frey testing, animals were euthanized to harvest bladder tissues. For euthanasia, rats were placed in a sealed chamber and exposed to 100% CO₂ delivered by gradual displacement at 30–70% of the chamber volume per minute. CO₂ flow was maintained for at least 1 min after respiratory arrest, and death was confirmed; cervical dislocation was then performed as a secondary method to ensure death, followed immediately by tissue collection (Table 2).

**Table 2:** Tissue collection conditions and Assays.

Portion (per rat, whole bladder tissues)	Approx. allocation	Processing	Assays	Notes
A	40–50%	Fix in 4% paraformaldehyde (PFA) → paraffin embedding → serial sectioning	H&E, Masson, IHC (BTK, tryptase), IF (BTK + tryptase)	One paraffin block can generate many serial sections for multiple stainings
B	5–10%	Cut into ~ 1 mm³ blocks → fix in 2.5% glutaraldehyde (4 °C) → 1% OsO₄ → embed	TEM (MC infiltration/degranulation)	TEM requires dedicated fixation; do not use PFA-fixed tissue
C	15–20%	Snap-freeze (liquid nitrogen) → store at − 80 °C → RNA extraction	RT-qPCR (BTK; ZO-1/Occludin/Claudin-1;Bcl-2/P21/c-Myc, etc.)	Keep as a separate frozen piece to avoid thaw–freeze cycles
D	15–20%	Snap-freeze → store at − 80 °C → protein extraction	Western blot (BTK; Bcl-2; P21; c-Myc; TJ proteins)	Use total protein lysate
E	15–20%	Snap-freeze → store at − 80 °C → PBS homogenization → centrifuge (e.g., 3000 rpm, 20 min)	ELISA (MPO, IL-1β, IL-6, TNF-α; HA, CS, DS, HS)	Work on ice; store supernatants at − 80 °C if not assayed immediately

All assays were performed using whole bladder tissues. For each rat, the excised bladder was evenly divided into the above portions for different downstream analyses.

### Experiment in vitro

**Fig. 1:**
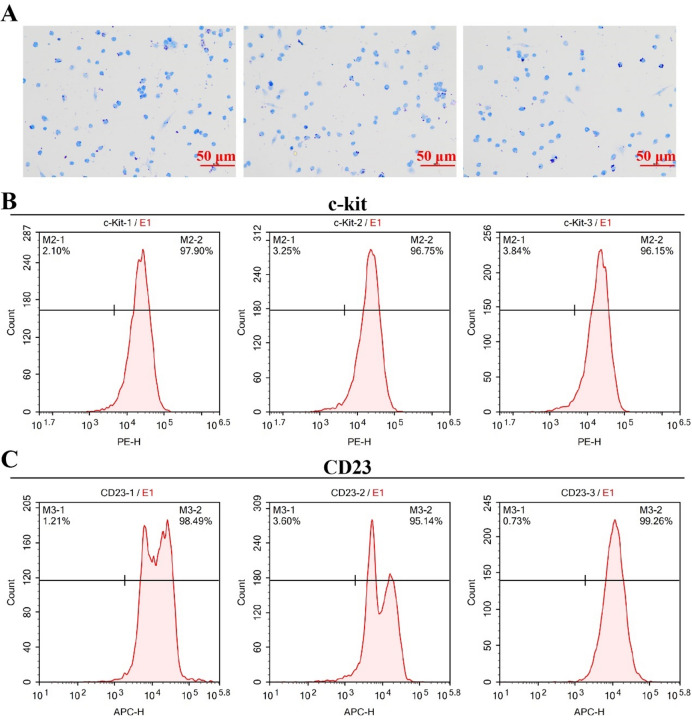
Identification of Mast cells (MCs) induced by Bone marrow mesenchymal stem cells (BMMSCs). **A**. Induced MCs Toluidine Blue (TB) staining, with a scale bar of 50 μm. **B-C**. Flow cytometry detects the proportion of MCs activation markers (c-kit, CD23) positive cells. The proportions of positive cells were 96.93 ± 0.89% (c-kit) and 97.63 ± 2.19% (CD23), respectively. As both values were > 90%, these results confirm successful MC induction.

Bone marrow mesenchymal stem cells (BMMSCs) were counted and seeded at 5 × 10^5 cells mL⁻¹ in 30 mL DMEM/F-12 supplemented with 100 ng mL⁻¹ recombinant human stem cell factor (rhSCF), 100 ng mL⁻¹ recombinant human interleukin-6 (rhIL-6), and 30 ng mL⁻¹ recombinant human interleukin-3 (rhIL-3); rhIL-3 was included only during the first week. After 1 week, the medium was replaced with fresh DMEM/F-12 containing 100 ng mL⁻¹ rhSCF and 50 ng mL⁻¹ rhIL-6, and cultures were maintained with weekly medium changes. From week 3 onward, non-adherent cells were transferred to new flasks at each change to remove adherent contaminants, and debris was discarded. Continuous induction for 7–10 weeks yielded mature mast cells (MCs)^10^. These cells were allowed to adhere to poly-L-lysine–coated coverslips, fixed, and stained with toluidine blue to visualize characteristic basophilic granules, and their immunophenotype was verified by flow-cytometric analysis of c-kit and CD23 surface expression (Fig. 1).

Human mast cells (MCs) and SV-HUC-1 human urothelial cells were cultured to investigate the role of BTK in MC activation and its regulatory effects on urothelial transmembrane barrier function. To establish an in vitro IC/BPS-like inflammatory Co-culture system, MCs were stimulated with varying concentrations of LL-37 (0.1, 1, 10, 20, and 100 µg/mL) for 12 h. Based on preliminary screening, 20 µg/mL was selected as the optimal concentration for subsequent experiments. MCs and SV-HUC-1 cells were then co-cultured in Transwell chambers to assess the impact of MC activation on epithelial barrier function. Before initiating co-culture, SV-HUC-1 cells were grown on Transwell chambers until a confluent monolayer was formed. Monolayer integrity was confirmed by TEER measurement: the resistance of a cell-free insert (blank) was subtracted from the total resistance and normalized to the membrane area (Ω·cm²). Co-culture was started only after TEER values were stable across consecutive measurements.

Six experimental groups were established (Table 3), BTK knockdown/overexpression was performed exclusively in mature MCs, whereas SV-HUC-1 cells were not genetically manipulated. Transduction was carried out after MC maturation, and transduction efficiency was confirmed prior to co-culture by flow cytometry. Only MCs with verified BTK knockdown/overexpression were used for subsequent LL-37 stimulation and Transwell co-culture experiments.

**Table 3:** Grouping of MCs Co-culture.

Groups	Co-culture (SV-HUC-1 cells co-cultured with LL-37–stimulated MCs)	KD-OV (MCs transduce with)
NC	None(SV-HUC-1 monoculture)	None
LL-37	Yes	None
LL-37 + KD-NC	Yes	knockdown negative-control (KD-NC) adenovirus
LL-37 + OV-NC	Yes	overexpression negative-control (OV-NC) adenovirus
LL-37 + KD-BTK	Yes	BTK-knockdown (KD-BTK) adenovirus
LL-37 + OV-BTK	Yes	BTK-overexpression (OV-BTK) adenovirus

### Molecular and functional experiments

MC proliferation was assessed using the Cell Counting Kit-8 (CCK-8) assay. Cell cycle progression and apoptosis rates were analyzed by flow cytometry, while cell invasive capacity was evaluated using Transwell chamber assays. MC degranulation was quantified by measuring tryptase and histamine secretion levels via ELISA, and the release of granule contents was further visualized by transmission electron microscopy (TEM).

To assess urothelial barrier function, ELISA was used to quantify the secretion of glycosaminoglycans (GAGs), including hyaluronic acid (HA), chondroitin sulfate (CS), dermatan sulfate (DS), and heparan sulfate (HS). The expression of tight junction proteins—ZO-1, Occludin, and Claudin-1—was measured by RT-qPCR and Western blotting. Transepithelial electrical resistance (TEER) assays were performed to evaluate barrier integrity, and patch-clamp electrophysiology was used to detect changes in membrane potential. Additionally, RT-qPCR and Western blotting were conducted to analyze the expression levels of BTK.

### Key experimental conditions

#### RT-qPCR

Real-time quantitative PCR was performed using FastKing RT Kit (with gDNase) for reverse transcription and Taq Pro Universal SYBR qPCR Master Mix for amplification. PCR was run under the following program: initial denaturation at 95 °C for 30 s; followed by 40–45 cycles of 95 °C for 10 s and 60 °C for 30 s. A melting-curve analysis was conducted at 95 °C for 10 s, 65 °C for 60 s, and 97 °C for 1 s. Relative mRNA expression was calculated using the 2^-ΔΔCt method with β-actin as the internal control.

#### Western blotting

Proteins were separated by SDS–PAGE under constant voltage (60 V until the dye front reached the resolving gel, then 90 V until completion). After electrophoresis, PVDF membranes were activated with methanol and wet-transferred; transfer current was adjusted according to protein molecular weight, and transfer was performed for 40 min. Membranes were briefly stained with Ponceau S to confirm transfer, blocked in 5% BSA for 2 h at room temperature, and washed with TBST. Membranes were incubated with primary antibodies at 4 °C overnight, washed with TBST (3 × 10 min), incubated with secondary antibodies for 2 h at room temperature, and washed again with TBST (3 × 10 min). Bands were visualized using enhanced chemiluminescence (ECL) and quantified by densitometry using ImageJ.

#### TEER measurement

Transepithelial electrical resistance (TEER) was measured after SV-HUC-1 monolayers were established in Transwell inserts. Before measurement, inserts were equilibrated in PBS for 30 min; the electrode was briefly rinsed/disinfected in 75% ethanol (10 s) and equilibrated in culture medium for 15 min. The electrode was then placed between the insert and the bottom well and stable resistance values were recorded in triplicate for each insert. TEER values were reported as Ω·cm², calculated using the insert area of 4.67 cm².

#### Patch-clamp electrophysiology (whole-cell current recording)

SV-HUC-1 cells were seeded at 5 × 10^4 cells per well in 12-well plates and cultured for 24 h.Patch-clamp recordings were performed after cells stabilized for ~ 10 min. Low-resistance glass microelectrodes were used to form a high-resistance seal, and cells were held at − 70 mV.Voltage steps from − 80 mV to + 80 mV were applied (0.5 s per step; 10 mV increment), and currents were recorded; each condition was recorded three times.

#### Fluorescent probe assay (Fluo-4 AM)

After treatment, cells were collected and centrifuged at 1000 rpm for 5 min, resuspended in Fluo-4 AM staining solution, and incubated at 37 °C for 30 min in the dark. Cells were washed with PBS, resuspended in buffer, incubated at 37 °C for an additional 5 min, and fluorescence signals were acquired by flow cytometry.

### Histological staining and quantification

#### Hematoxylin and eosin (H&E) staining

Paraffin sections were deparaffinized, rehydrated through graded ethanol, and stained with hematoxylin and eosin to evaluate overall bladder morphology, including epithelial integrity, inflammatory cell infiltration, edema/hemorrhage, and bladder wall structure. Scale bars: 100 μm and 50 μm.

#### Masson’s trichrome staining & Quantification of collagen deposition

Masson’s trichrome–stained rat bladder sections were imaged at 10× magnification under identical microscope and camera settings. The region of interest (ROI) was defined as the entire bladder wall, while the luminal space and areas with folds, tearing, or other artifacts were excluded. Collagen fibers (blue) and muscle fibers (red) were evaluated on the same sections, and collagen deposition was quantified as the percentage of collagen-positive area within the ROI. Images were analyzed using ImageJ: collagen staining was separated and segmented by color-based separation and thresholding, with the same segmentation criteria applied across all groups. Image quantification was performed in a blinded manner.

#### Immunohistochemistry (IHC) staining

Paraffin-embedded bladder sections were baked, deparaffinized in xylene, and rehydrated through graded ethanol. Sections were washed with PBS and subjected to antigen retrieval in citrate buffer (pH 6.0) using microwave heating. Endogenous peroxidase activity was blocked with 3% hydrogen peroxide, followed by serum blocking. Sections were incubated with primary antibodies against BTK and tryptase overnight at 4 °C, then with secondary antibodies at room temperature. Signals were developed with DAB, counterstained with hematoxylin, dehydrated, mounted, and imaged under a light microscope.

#### Immunofluorescence (IF) staining

Paraffin sections were baked, deparaffinized, and rehydrated, followed by PBS washes. Antigen retrieval was performed in citrate buffer (pH 6.0) using microwave treatment. After serum blocking, sections were incubated with primary antibodies overnight at 4 °C in the dark, followed by fluorophore-conjugated secondary antibodies. Sections were mounted with antifade medium containing DAPI and examined under a fluorescence microscope.

#### Mast cell counting (tryptase IHC)

Tryptase-positive mast cells were quantified on bladder tissue sections following tryptase IHC staining. Images were acquired under light microscopy at 10× magnification using identical acquisition settings. The region of interest (ROI) was defined as the full-thickness bladder wall (excluding the lumen), and areas with folds, tearing, poor staining, or non-specific staining artifacts as well as section edges were excluded. Tryptase-positive mast cells were identified by specific brown cytoplasmic DAB staining and were counted using ImageJ/Fiji (NIH) with manual verification. For each animal, three non-adjacent sections were analyzed, and in each section five non-overlapping fields were selected. Counts were expressed as cells per field, and the mean value per animal (average across fields/sections) was used for statistical analysis. Cell counting was performed by two investigators blinded to group allocation.

### Statistics

All data were analyzed and visualized using GraphPad Prism 8.0 software. Quantitative data with normal distribution were expressed as mean ± standard deviation (SD). Comparisons between two groups were performed using the Student’s *t*-test, while one-way analysis of variance (ANOVA) was used for comparisons among multiple groups. Tukey’s post hoc test was applied for pairwise comparisons between multiple groups. A *P* value < 0.05 was considered statistically significant. For animal experiments, n denotes the number of rats per group (*n* = 6). For in vitro experiments, n denotes the number of independent biological replicates (3 independent experiments), unless otherwise stated. Unless otherwise specified, error bars indicate SD.

## Results

### Animal model

#### Validation of AAV-mediated BTK overexpression/knockdown in bladder tissues

**Fig. 2:**
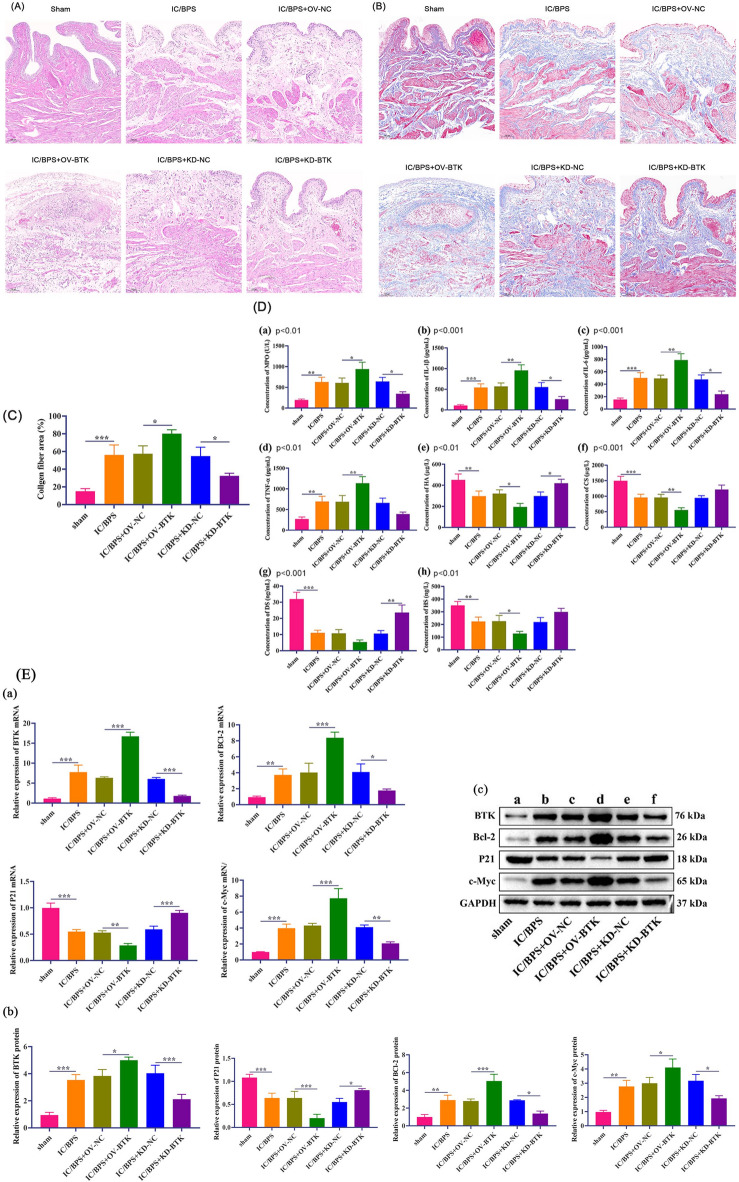
BTK modulation in the LL-37 IC/BPS rat model alters bladder pathology, inflammatory cytokine production, and urothelial GAG content. **A.** Rat Bladder Tissue (H&E Staining); **B-C**. Rat Bladder Tissue (Masson Staining) & Statistics of collagen fibers in bladder tissue (Blue: collagen fiber, red: muscle fiber, ROI: Whole bladder wall); **D**. Concentrations of MPO (a), IL-1β (b), IL-6 (c), and TNF-α (d) in bladder tissues, as well as HA (e), CS (f), DS (g), and HS (h) in urinary tract tissues detected by ELISA. *P < 0.05, **P < 0.01, ***P < 0.001. **E**. RT-qPCR(a) and Western blot(b-c) analysis of BTK expression in rat bladder tissues. (Non-contiguous lanes were assembled from the same blot; vertical lines indicate where lanes were spliced.). *Data are presented as mean ± SD (*n* = 6 rats per group). Error bars indicate SD. Statistical analysis was performed using one-way ANOVA followed by Tukey’s post hoc test. **Animal experimental groups: Sham (intravesical normal saline + normal saline control), IC/BPS (LL-37 only), IC/BPS + OV-NC (LL-37 + AAV negative control for overexpression), IC/BPS + OV-BTK (LL-37 + AAV-BTK overexpression), IC/BPS + KD-NC (LL-37 + AAV negative control for knockdown), and IC/BPS + KD-BTK (LL-37 + AAV-BTK knockdown).

- RT-qPCR analysis showed that BTK mRNA expression in bladder tissues was significantly increased in LL-37–induced IC/BPS rats compared with the sham group (*P* < 0.001; Fig. 2E(a)). Compared with the OV-NC group, the OV-BTK group exhibited a marked increase in BTK mRNA levels (*P* < 0.001). Conversely, BTK mRNA expression was significantly suppressed in the KD-BTK group (*P* < 0.001; Fig. 2E(a)).
- Western blotting further confirmed that BTK protein levels were significantly higher in the IC/BPS group than in the sham group (*P* < 0.001; Fig. 2E(b–c)). No significant difference in BTK protein expression was observed between the OV-NC or KD-NC groups and the IC/BPS group (*P* > 0.05). BTK protein levels were increased in the OV-BTK group compared with the OV-NC group (*P* < 0.05), whereas BTK knockdown produced the opposite effect (*P* < 0.001; Fig. 2E(b–c)).

These results indicate that BTK upregulation in the LL-37–induced IC/BPS model is accompanied by increased expression of pro-survival/proliferation markers (Bcl-2 and c-Myc) and decreased expression of the cell-cycle inhibitor P21, whereas BTK knockdown reverses these molecular changes.3.1.2 Pathological effect of BTK expression on bladder tissue of IC/BPS rats.

- Following euthanasia, bladder tissues were harvested from rats for histological analysis. Rat bladder tissue Hematoxylin and eosin (H&E) staining (Fig. 2A) showed that bladder tissues in the sham group exhibited intact architecture, thick mucosa, and abundant smooth muscle fibers, with no signs of inflammatory cell infiltration. In contrast, the IC/BPS group displayed typical inflammatory features, including submucosal hemorrhage, pronounced interstitial edema, and marked inflammatory cell infiltration. In the BTK overexpression group, inflammatory infiltration was further aggravated, with partial epithelial loss and increased microvascular density in the interstitium. Compared with the KD-NC group, the BTK knockdown group showed reduced inflammatory infiltration and a thickened bladder wall;
- Masson’s trichrome staining (Fig. 2B–C) revealed well-organized and abundant smooth muscle fibers in the sham group. In the IC/BPS group, there was significant collagen fiber deposition, which was markedly higher than in the sham group (*P* < 0.001). BTK overexpression further increased collagen accumulation and disrupted smooth muscle fiber organization (*P* < 0.05). In contrast, collagen fiber content was reduced and fibrosis was alleviated in the BTK knockdown group compared to KD-NC (*P* < 0.05). These findings indicate that BTK knockdown mitigates LL-37-induced bladder inflammation and fibrosis in IC/BPS rats.

#### Regulation of BTK on bladder inflammation and urinary epithelial GAGs in IC/BPS rats

- Inflammatory markers including myeloperoxidase (MPO), interleukin-1β (IL-1β), IL-6, and tumor necrosis factor-α (TNF-α) in bladder tissues were quantified by ELISA (Fig. 2D(a–d)). Compared to the sham group, levels of MPO (Fig. 2D(a)), IL-1β (Fig. 2D(b)), IL-6 (Fig. 2D(c)), and TNF-α (Fig. 2D(d)) were significantly elevated in the IC/BPS group. These inflammatory mediators were further upregulated in the BTK overexpression group (MPO: *P* < 0.05; IL-1β: *P* < 0.01; IL-6: *P* < 0.01; TNF-α: *P* < 0.01). Conversely, BTK knockdown significantly attenuated LL-37-induced upregulation of these inflammatory markers (MPO, IL-1β, IL-6: *P* < 0.05; TNF-α: *P* < 0.05).
- ELISA results of glycosaminoglycan (GAG) levels in bladder tissues showed that concentrations of hyaluronic acid (HA), chondroitin sulfate (CS), dermatan sulfate (DS), and heparan sulfate (HS) were significantly reduced in the IC/BPS group compared to the sham group (HA: *P* < 0.01, Fig. 2D(e); CS: Fig. 2D(f); DS: Fig. 2D(g); HS: Fig. 2D(h)). BTK overexpression further decreased HA (*P* < 0.05), CS (*P* < 0.01), DS, and HS (*P* < 0.05), while BTK knockdown significantly increased the levels of HA (*P* < 0.05), CS, DS (*P* < 0.01), and HS compared to the respective controls.

#### Regulation of BTK on urinary epithelial barrier in IC/BPS rats

**Fig. 3:**
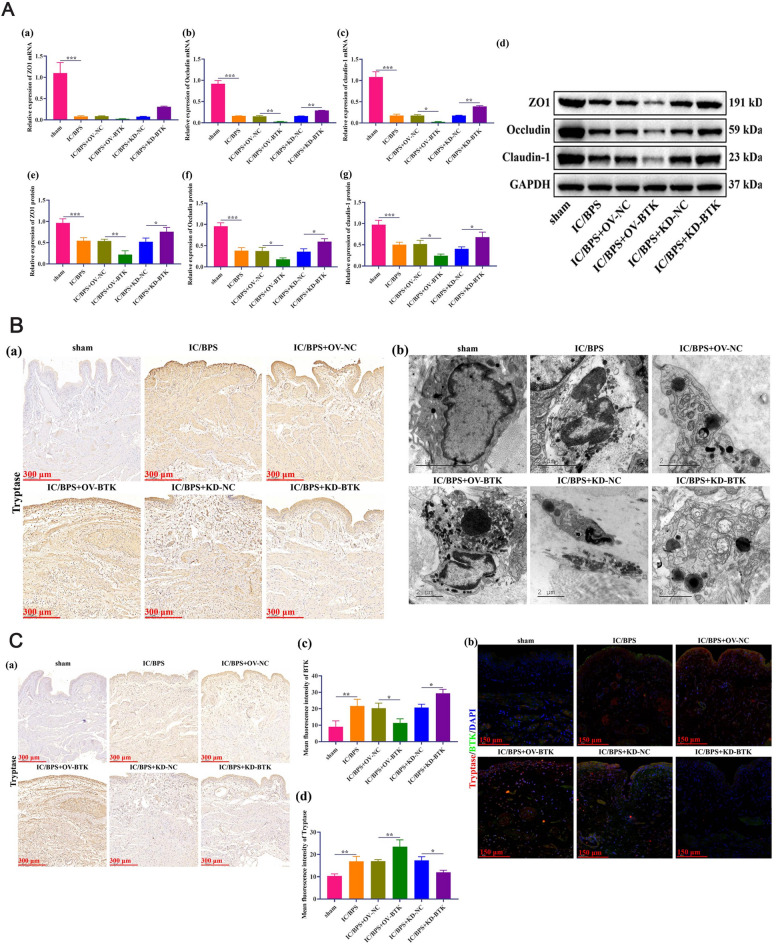
In LL-37–induced IC/BPS rats, BTK upregulation is associated with mast cell degranulation and loss of urothelial tight-junction proteins, while BTK knockdown mitigates these effects. **A**. (a)-(c) Expression of **tight-junction proteins** (TJ)-Related Markers ZO-1, Occludin, and Claudin-1 in Bladder Tissue by RT-qPCR. (d)-(g) Expression of TJ-Related Markers ZO-1, Occludin, and Claudin-1 in Bladder Tissue by Western blot (Non-contiguous lanes were assembled from the same blot; vertical lines indicate where lanes were spliced.); **B**. (a): Immunohistochemical (IHC) Staining of Tryptase-Positive Cells in Bladder Tissue (Brown-yellow positive cells indicated; 10× magnification). (b): Transmission Electron Microscopy (TEM) Analysis of Mast Cell (MC) Degranulation in Rat Bladder Tissue. **C**. (a): The positive rate of BTK in bladder tissue cells was detected by IHC staining (the positive cells were brown) (10 ×). (b): IF staining was used to detect the fluorescence intensity of BTK and tryptase in bladder tissue (20 ×). (c): Fluorescence intensity statistics of BTK. (d): Fluorescence intensity statistics of tryptase. *P < 0.05, **P < 0.01. *Data are presented as mean ± SD (*n* = 6 rats per group). Error bars indicate SD. Statistical analysis was performed using one-way ANOVA followed by Tukey’s post hoc test. **Animal experimental groups: Sham (intravesical normal saline + normal saline control), IC/BPS (LL-37 only), IC/BPS + OV-NC (LL-37 + AAV negative control for overexpression), IC/BPS + OV-BTK (LL-37 + AAV-BTK overexpression), IC/BPS + KD-NC (LL-37 + AAV negative control for knockdown), and IC/BPS + KD-BTK (LL-37 + AAV-BTK knockdown).

- The expression of tight junction (TJ)-related markers ZO-1, Occludin, and Claudin-1 in bladder tissues was evaluated by RT-qPCR (Fig. 3A(a–c)) and Western blotting (Fig. 3A(d–g)). Compared with the sham group, mRNA and protein levels of ZO-1 (*P* < 0.001, Fig. 3(a, e)), Occludin (*P* < 0.001, Fig. 3A (b, f)), and Claudin-1 (*P* < 0.001, Fig. 3A (c, g)) were significantly decreased in the IC/BPS group.
- Compared with the OV-NC group, BTK overexpression was associated with lower ZO-1 and occludin expression (*P* < 0.01) and reduced claudin-1 expression (*P* < 0.05) at both the mRNA and protein levels. Conversely, compared with the KD-NC group, BTK knockdown was associated with higher expression of ZO-1, occludin (*P* < 0.01), and claudin-1 (*P* < 0.01).
- These findings suggest that LL-37 stimulation induces bladder inflammation, disrupts the GAG layer of the bladder, and impairs urothelial barrier function. Inhibition of BTK attenuates LL-37-induced inflammation and preserves urothelial barrier integrity.

#### Regulation of BTK expression on degranulation of MCs in bladder tissue of IC/BPS rats

- Immunohistochemical (IHC) staining was performed to detect tryptase-positive cells in bladder tissue sections (Fig. 3B(a)). Compared with the sham group, the number of tryptase-positive cells was significantly increased in the IC/BPS group. The BTK overexpression group showed more tryptase-positive cells than the OV-NC group, while the BTK knockdown group exhibited a reduction in tryptase-positive cells.
- Transmission electron microscopy (TEM) was used to evaluate mast cell (MC) degranulation in bladder tissues (Fig. 3B(b)). MCs in the bladder exhibited diverse morphologies. In the IC/BPS group, cytoplasmic granules—spherical or ovoid in shape—were observed being released into the extracellular space. These granules varied in size and were membrane-bound. The BTK overexpression group displayed increased extracellular granule release compared to controls, whereas fewer granules were observed in the BTK knockdown group.
- These results indicate that BTK overexpression promotes mast cell activation and degranulation in the bladder tissue of IC/BPS rats, while BTK knockdown reduces mast cell activation in this pathological context.

#### Expression of BTK in bladder tissue of IC/BPS rats

- Immunohistochemical (IHC) staining was used to assess the distribution and abundance of BTK immunoreactivity within the bladder wall in rat bladder tissues (Fig. 3C(a)). Compared to the sham group, the IC/BPS group showed a significant increase in BTK-positive cells across the bladder tissue. The number of BTK-positive cells was further elevated in the BTK overexpression group compared to the OV-NC group, whereas BTK knockdown led to a marked reduction in BTK-positive cells.
- Additionally, immunofluorescence (IF) staining was performed to evaluate BTK signals and their co-localization with tryptase-positive mast cells in bladder tissues (Fig. 3C(b–d)). Consistent with the IHC findings, fluorescence intensities of both BTK and tryptase were significantly higher in the IC/BPS group compared to the sham group (*P* < 0.01). BTK overexpression further enhanced the fluorescence intensities of BTK (*P* < 0.05) and tryptase (*P* < 0.01), while BTK knockdown resulted in a significant reduction in their fluorescence signals (*P* < 0.05).

### BTK regulates MCs activation

#### Expression of BTK in MCs and its regulation on SV-HUC-1 membrane ions

**Fig. 4:**
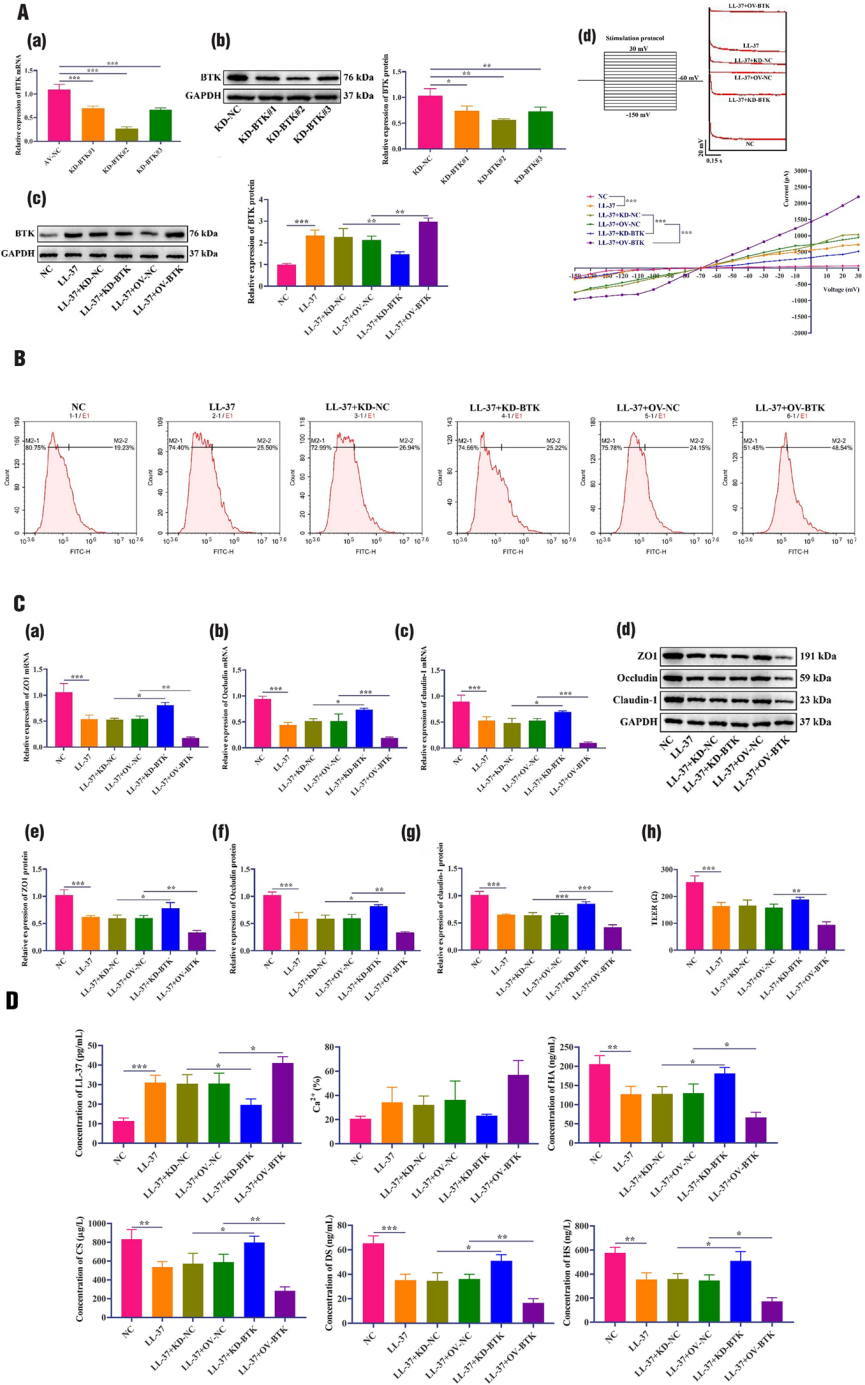
BTK modulates LL-37–activated mast cells to alter SV-HUC-1 electrophysiology, reduce glycosaminoglycans (GAGs) and tight-junction proteins, and impair barrier function, while BTK knockdown mitigates these effects. **A**. Expression of BTK in MCs and its regulation on membrane ions of sv-huc-1 cells (Non-contiguous lanes were assembled from the same blot; vertical lines indicate where lanes were spliced.)(a): The expression of BTK mRNA was analyzed by RT qPCR; (b) and (c): the relative expression of BTK protein was detected by Western blot; (d): The whole cell current was measured by patch clamp technique. *P < 0.05, **P < 0.01, ***P < 0.001. **B**. Detection of membrane changes of sv-huc-1 cells by calcium channel fluorescent probe. **C**. BTK regulates urothelial barrier injury by activating MCs (Non-contiguous lanes were assembled from the same blot; vertical lines indicate where lanes were spliced.). The mRNA expressions of ZO1 (a), occludin (b) and claudin-1 (c) were detected by RT qPCR. (d): The relative expressions of ZO1 (e), occludin (f) and claudin-1 (g) proteins were detected by Western blot. (h): TEER detection of sv-huc-1 cells* *P* < 0.05, **P < 0.01, ***P < 0.001. **D**. ELISA kits were used to measure the levels of LL-37, HA (D), CS (E), DS (F), and HS (G) in the culture supernatants of SV-HUC-1 cells in each group. *P < 0.05, **P < 0.01, ***P < 0.001. *Data are presented as mean ± SD from 3 independent experiments. Error bars indicate SD. Statistical analysis was performed using one-way ANOVA followed by Tukey’s post hoc test. **In vitro experimental groups: (1) NC, SV-HUC-1 monoculture; (2) LL-37, SV-HUC-1 co-cultured with MCs stimulated with 20 µg/mL LL-37; (3) LL-37 + KD-NC, co-culture with LL-37–stimulated MCs transduced with KD-NC adenovirus; (4) LL-37 + OV-NC, co-culture with LL-37–stimulated MCs transduced with OV-NC adenovirus; (5) LL-37 + KD-BTK, co-culture with LL-37–stimulated MCs transduced with KD-BTK adenovirus; (6) LL-37 + OV-BTK, co-culture with LL-37–stimulated MCs transduced with OV-BTK adenovirus. BTK modulation was performed in MCs; SV-HUC-1 cells were not genetically manipulated.

- To determine the most effective BTK knockdown target, three BTK-targeting shRNA adenoviral vectors (KD-BTK#1, #2, #3) were transfected into 293T cells (Human Embryonic Kidney 293T). 293T cells were selected because of their high transfection efficiency and robust exogenous gene expression, enabling rapid and reliable comparison of multiple candidate targets. Candidate BTK-targeting vectors were transfected into 293T cells, and knockdown efficiency was evaluated by RT-qPCR and Western blotting based on reductions in BTK mRNA and protein levels. The construct exhibiting the strongest inhibitory effect was selected as the optimal target for use in the formal transfection experiments. After 48 h, strong fluorescence indicated successful transfection, and RT-qPCR showed significantly reduced BTK mRNA in all knockdown groups versus KD-NC (*P* < 0.001, Fig. 4A(a)). Western blot confirmed reduced BTK protein expression (*P* < 0.05, Fig. 4A(b)), with KD-BTK#2 being the most efficient.
- MCs were then transduced with KD-NC, KD-BTK#2 (KD-BTK), OV-NC, or OV-BTK and stimulated with 20 µg/mL LL-37. LL-37 significantly increased BTK expression in MCs (*P* < 0.001, Fig. 4A(c)); BTK was significantly reduced in KD-BTK (*P* < 0.01) and increased in OV-BTK (*P* < 0.01) compared to their respective controls.
- To assess barrier function, SV-HUC-1 cells were co-cultured with treated MCs. LL-37-treated MCs increased SV-HUC-1 whole-cell currents (*P* < 0.001). Co-culture with LL-37 + KD-BTK MCs reduced current versus LL-37 + KD-NC (*P* < 0.001), while LL-37 + OV-BTK further increased current compared to LL-37 + OV-NC (*P* < 0.001, Fig. 4B).

#### BTK regulates MCs activation and affects gags levels in SV-HUC-1 cells

- ELISA revealed significantly elevated LL-37 levels in SV-HUC-1 supernatants co-cultured with LL-37-stimulated MCs compared to control (*P* < 0.001). BTK knockdown significantly reduced LL-37 levels versus KD-NC (*P* < 0.05), while BTK overexpression increased them (*P* < 0.05).
- ELISA of SV-HUC-1 supernatants showed that co-culture with LL-37-stimulated MCs significantly reduced HA (*P* < 0.01), CS (*P* < 0.01), DS (*P* < 0.001), and HS (*P* < 0.01) levels. BTK knockdown significantly increased these GAGs compared to KD-NC (*P* < 0.05), while overexpression significantly decreased them compared to OV-NC (HA: *P* < 0.05; CS: *P* < 0.01; DS: *P* < 0.01; HS: *P* < 0.05).

#### BTK regulates MCs activation and affects urothelial barrier

- RT-qPCR and Western blot analyses were performed to evaluate the expression levels of tight junction (TJ)-related genes ZO-1, Occludin, and Claudin-1 in SV-HUC-1 cells. As shown in Fig. 4C(a–c), SV-HUC-1 cells co-cultured with LL-37-stimulated MCs exhibited significantly decreased mRNA expression of ZO-1 (*P* < 0.001), Occludin (*P* < 0.001), and Claudin-1 (*P* < 0.001) compared with the SV-HUC-1 monoculture group. Knockdown of BTK in LL-37-stimulated MCs significantly restored the mRNA expression of ZO-1 (*P* < 0.05), Occludin (*P* < 0.05), and Claudin-1 (*P* < 0.05) in SV-HUC-1 cells. Conversely, BTK overexpression in LL-37-stimulated MCs further suppressed ZO-1 (*P* < 0.01), Occludin (*P* < 0.001), and Claudin-1 (*P* < 0.001) expression compared to the LL-37-only co-culture group.
- Western blot results were consistent with RT-qPCR findings (Fig. 4C(d–g)). Protein levels of ZO-1 (*P* < 0.001, Fig. 4C(e)), Occludin (*P* < 0.001, Fig. 4C(f)), and Claudin-1 (*P* < 0.001, Fig. 4C (g)) were significantly decreased in SV-HUC-1 cells co-cultured with LL-37-treated MCs. BTK knockdown led to a significant increase in ZO-1 (*P* < 0.05), Occludin (*P* < 0.05), and Claudin-1 (*P* < 0.001) protein expression, while BTK overexpression further suppressed these levels of protein compared with the OV-NC group (ZO-1: *P* < 0.01; Occludin: *P* < 0.01; Claudin-1: *P* < 0.001).
- Transepithelial electrical resistance (TEER) measurements of SV-HUC-1 monolayers were performed to assess barrier integrity (Fig. 4C(h)). Co-culture with LL-37-stimulated MCs significantly reduced TEER values compared to the monoculture group (*P* < 0.001). BTK knockdown slightly increased TEER, while BTK overexpression led to a marked reduction in TEER values (*P* < 0.01).
- Taken together, these findings suggest that BTK promotes LL-37-induced MC activation, which in turn disrupts the urothelial barrier. Conversely, BTK knockdown attenuates MC activation and helps preserve urothelial barrier function.

### Figures, tables and schemes

#### Main experimental reagents and manufacturers

**Table:** 

Human recombinant IL-3 and human recombinant stem cell factor SCF	Gibco™, Thermo Fisher Scientific (Waltham, MA, USA)
SV-HUC-1 cells (human urothelial cell line), Human bone marrow mesenchymal stem cells (HBMMSC)	Shanghai Zhongqiao Xinzhou Biotechnology Co., Ltd. (Shanghai, China)
Annexin V-FITC/PI Apoptosis Detection Kit	Absin Bioscience Inc. (Shanghai, China)
CCK-8 Assay Kit	Beyotime Biotechnology (Shanghai, China)
Transwell Chamber	Corning Incorporated (USA)
Cell Cycle Detection Kit	Elabscience Biotechnology Co., Ltd. (Wuhan, China)
Human Trypsin and Histamine ELISA Kits	MLBIO – Shanghai Enzyme-linked Biotechnology Co., Ltd.
FastKing RT Kit (With gDNase), FastKing First-Strand cDNA Synthesis Kit	TIANGEN Biotech Co., Ltd. (Beijing, China)
Taq Pro Universal SYBR qPCR Master Mix	Vazyme Biotech Co., Ltd. (Nanjing, China)
Protease Inhibitor Cocktail, PhosSTOP Phosphorylation Protease Inhibitor	Roche Diagnostics (Switzerland)
BTK Antibody (DF6472)	Affinity Biosciences Inc. (USA)
Mast Cell Tryptase (Ab2378), Pre-adsorbed Goat Anti-Mouse IgG H&L (CY3) (ab97035)	Abcam plc (UK)
DyLight 488 Labeled Goat Anti-Rabbit IgG (H + L) (5230 − 0385)	KPL (SeraCare Life Sciences, USA)
Antibodies: Bcl-2 (bsm-33411 M), p21 (bs-55160R), ZO-1 (bs-1329R), Occludin (bs-10011R), Claudin-1 (bs-1428R)	Bioss Antibodies (Beijing, China)
Antibodies: c-Myc (18583 S), Secondary Antibodies (7074, 7076)	Cell Signaling Technology, Inc. (USA)
GAPDH Antibody (P30008M)	Abmart Inc. (Shanghai, China)

## Discussion

In this study, we combined an LL-37–induced IC/BPS rat model with an in vitro Transwell co-culture system to investigate the role of BTK in mast cell (MC)–mediated urothelial barrier dysfunction. In vivo, our model reproduced key pathological features implicated in IC/BPS—mast cell–associated inflammation and urothelial barrier injury—manifested by inflammatory infiltration, increased collagen deposition, elevated pro-inflammatory mediators, enhanced tryptase-positive mast cell signals, and loss of tight-junction–associated proteins indicative of impaired barrier integrity. This phenotype was further supported by pain-related behavioral validation using the von Frey test. Importantly, AAV-mediated modulation of BTK provided bidirectional functional evidence: BTK upregulation exacerbated bladder inflammation, mast cell degranulation, and urothelial damage, whereas BTK knockdown mitigated these pathological changes. This “worsening-by-upregulation and rescue-by-inhibition” pattern suggests that BTK is not merely a correlated marker but may function upstream to amplify mast cell–driven inflammatory cascades and promote GAG/TJ disruption in an IC/BPS-like context. Prior bioinformatic analyses and studies using human IC/BPS specimens have reported aberrant mast-cell signatures in the bladder wall and dysregulation of BTK^11^. These findings provided a rationale to use a more controllable in vitro setting to dissect the direct effects of LL-37 on mast-cell activation and the functional consequences of LL-37–activated mast cells on urothelial barrier integrity. Under these conditions, we obtained the following results: in vitro, SV-HUC-1 cells co-cultured with LL-37–stimulated MCs exhibited increased whole-cell currents and intracellular calcium concentrations, along with reduced GAGs (HA, CS, DS, and HS) and TJ proteins (ZO-1, occludin, and claudin-1). These barrier-disruptive changes were further aggravated by BTK overexpression in MCs and attenuated by BTK knockdown, supporting a regulatory role of BTK in MC-driven urothelial barrier disruption.

In recent years, in vitro co-culture systems have been increasingly used to dissect how MC degranulation and granule-stored mediators influence neighboring cells. For example, co-culture with MCs promotes gastric cancer cell proliferation, invasion, migration, and resistance to H₂O₂-induced apoptosis^12^, and MCs enhance human lung fibroblast contractility in a time- and concentration-dependent manner^13^. Within the IC/BPS field, accumulating evidence suggests that MCs may contribute to disease progression, and their impact on epithelial barrier function is well documented^14^. In a mouse model of irritable bowel syndrome, increased paracellular permeability and enhanced MC degranulation were accompanied by morphological disruption of the colonic epithelial barrier and downregulation of TJ proteins such as ZO-2 and occludin^14^, providing a conceptual framework for interpreting our bladder findings.

Mechanistically, MC-derived mediators can trigger epithelial barrier dysfunction. Histamine, tryptase, and prostaglandin D₂ (PGD₂) have been implicated in altered epithelial secretion and permeability, while other MC-derived products may directly impair epithelial integrity^15^. Notably, tryptase and chymase can proteolytically cleave TJ proteins (e.g., claudins) and junctional adhesion molecule A (JAM-A)^16^. Groschwitz et al. further showed that MC-derived tryptase activates protease-activated receptor 2 (PAR2), leading to ERK1/2 phosphorylation, perijunctional F-actin reorganization, and increased epithelial permeability^16^. Consistent with the broad effects of MC degranulation on TJ integrity across epithelial systems^17,18^, our results suggest that BTK may act upstream to potentiate LL-37–induced MC activation/degranulation, thereby amplifying mediator-driven disruption of the urothelial GAG layer and TJ complex. However, our current data do not identify the specific granule-derived mediators responsible for TJ/GAG alterations, and further studies are needed to delineate these pathways.

Moreover, the patch-clamp findings—together with elevated intracellular calcium signals—indicate electrophysiological remodeling of SV-HUC-1 cells during MC-mediated barrier impairment. The ion channels responsible for these changes remain unknown and warrant targeted investigation using channel-specific inhibitors and genetic perturbation.

Clinically, BTK is a key regulator in multiple immune cell types, and BTK inhibitors have shown anti-inflammatory potential in various disease settings^19^. Our in vivo and in vitro data collectively indicate that BTK activity is closely associated with inflammatory severity and urothelial barrier disruption in an IC/BPS-like context, suggesting BTK as a promising therapeutic target and potential biomarker candidate; nevertheless, further validation in human tissues and larger cohorts is required before clinical translation.

We acknowledge that stimulating mast cells with LL-37 in vitro and co-culturing them with urothelial cells does not fully recapitulate the complexity of interstitial cystitis/bladder pain syndrome (IC/BPS), and this reductionist system warrants further validation. Likewise, although the intravesical LL-37 challenge reproduced several key IC/BPS-relevant features—including mast cell–associated inflammation, urothelial barrier injury (GAG/TJ impairment), extracellular matrix remodeling—the model primarily represents an inducible inflammatory/barrier-injury process and may not fully capture the chronicity, heterogeneity (e.g., Hunner vs. non-Hunner phenotypes), or the full spectrum of symptom dimensions observed in patients. Future studies incorporating long-term/relapsing paradigms, comprehensive urodynamic and behavioral assessments, cell-type–restricted genetic approaches, and validation in human tissues and larger cohorts will be essential to strengthen the translational relevance of BTK as a therapeutic target and biomarker candidate in IC/BPS. Despite these limitations, our study has several strengths. First, the combined in vivo–in vitro design provides convergent evidence that links BTK activity to mast cell–driven urothelial barrier dysfunction across complementary model systems. Second, AAV-mediated BTK upregulation versus knockdown offers functional support beyond correlative observations and strengthens causal inference regarding BTK in IC/BPS-like pathology. Collectively, these strengths provide a framework for future translational studies targeting BTK in IC/BPS.

## Conclusions

BTK overexpression promotes mast cell proliferation, invasion, and degranulation, leading to impaired expression of glycosaminoglycans (GAGs) and tight junction (TJ) proteins in SV-HUC-1 cells, thereby compromising urothelial barrier function. This may represent one of the mechanisms by which BTK contributes to the pathogenesis of IC/BPS. In contrast, BTK knockdown attenuates MC activation and alleviates their detrimental effects on epithelial barrier integrity.

## Supplementary Information

Below is the link to the electronic supplementary material.

Supplementary Material 1

Supplementary Material 2

Supplementary Material 3

Supplementary Material 4

Supplementary Material 5

Supplementary Material 6

Supplementary Material 7

Supplementary Material 8

Supplementary Material 9

Supplementary Material 10

Supplementary Material 11

Supplementary Material 12

Supplementary Material 13

Supplementary Material 14

Supplementary Material 15

Supplementary Material 16

Supplementary Material 17

Supplementary Material 18

Supplementary Material 19

## Data Availability

Data is provided within the manuscript or supplementary information files.
