# Supplementary material for: Role of Bruton’s Tyrosine Kinase in mast cell driven urothelial barrier injury in an LL-37 induced model of interstitial cystitis
Source: Sci Rep. 2026 Apr 30;16:20181. doi: 10.1038/s41598-026-50443-z (PMC13323726; doi:10.1038/s41598-026-50443-z)
Supplement: Supplementary file 1 — Supplementary Material 1 [file 41598_2026_50443_MOESM1_ESM.docx]

**Effects of different concentrations of LL-37 on the phenotype of MCS**

**1.** **LL-37 promotes the proliferation and cell cycle progression of MCS**

In preliminary experiments, an in vitro IC/BPS cell model was established by treating mast cells (MCs) with various concentrations of LL-37 (0, 0.1, 1, 10, 20, and 100 μg/mL). Cell proliferation was assessed using the Cell Counting Kit-8 (CCK-8) assay. As shown in Figure 1A, MC proliferation increased with rising LL-37 concentrations, and significant differences were observed at 10, 20, and 100 μg/mL compared to the control group (*P* < 0.05). The proliferative effects at 20 μg/mL and 100 μg/mL were comparable and both statistically significant (*P* < 0.01).

Cell cycle progression was evaluated by flow cytometry. As shown in Figure 1(B–C), LL-37 treatment promoted the transition of MCs from G1 to S phase. Significant changes were detected in the 1, 10, 20, and 100 μg/mL treatment groups compared to the control (*P* < 0.001).

**2.** **LL-37 stimulation promotes MCS invasion and reduces apoptosis**

The invasive capacity of MCs was evaluated using Transwell chambers. As shown in Figure 2(A–B), MCs exposed to LL-37 exhibited greater invasive potential compared to untreated controls. Significant differences in invasion were observed in the 10, 20, and 100 μg/mL LL-37 treatment groups compared with the untreated group (*P* < 0.001).

Apoptosis was assessed by flow cytometry using Annexin V-FITC/PI double staining (Figure 2(C–D)). The apoptosis rate of MCs gradually decreased with increasing concentrations of LL-37. Statistically significant reductions in apoptosis were detected in the 10, 20, and 100 μg/mL LL-37 treatment groups compared to the untreated group (*P* < 0.01).

**3. LL-37 stimulation promotes degranulation of MCS**

The concentrations of tryptase and histamine were measured using ELISA kits (Figure 3(A–B)). With increasing LL-37 concentrations, both tryptase (Figure 3A) and histamine (Figure 3B) levels in MCs increased significantly. Notably, the concentrations of these mediators in the 20 μg/mL and 100 μg/mL LL-37 treatment groups were comparable (*P* < 0.001).

Transmission electron microscopy (TEM) was used to observe granule release in MCs following LL-37 stimulation (Figure 3C). LL-37 exposure induced the extracellular release of granule contents, indicating enhanced mast cell activation.

Taken together, LL-37 exposure promoted MC proliferation, G1-to-S phase cell cycle progression, invasion, and degranulation, while reducing apoptosis and inducing overall MC activation. Given the similar biological effects observed between 20 μg/mL and 100 μg/mL LL-37 treatments, 20 μg/mL was selected as the optimal concentration for subsequent experiments.

**4. LL-37 induces MCS to construct IC/PBS model**

The optimal concentration of LL-37 for inducing mast cell (MC) activation in IC/BPS models remains unclear. To determine the most effective concentration, we stimulated MCs with 0.1, 1, 10, 20, and 100 μg/mL LL-37 for 12 hours. The results showed that increasing LL-37 concentrations promoted MC proliferation, cell cycle progression, and invasive capacity, and significantly enhanced degranulation. Notably, the biological effects of 20 μg/mL and 100 μg/mL LL-37 on MCs were comparable.

Previous studies have reported that treatment of primary human urothelial cells with LL-37 at concentrations of 0, 0.3, 1, 3, 10, 25, 100, and 250 μM resulted in a concentration-dependent increase in apoptosis. However, no significant differences in apoptosis rates were observed between the 25, 100, and 250 μM treatment groups[206].

Our study is the first to demonstrate that LL-37 can induce mast cell (MC) activation and promote degranulation. Furthermore, BTK knockdown was found to inhibit LL-37–induced MC proliferation and degranulation. These findings suggest that BTK may serve as a key regulatory target for suppressing MC degranulation in IC/BPS and provide a theoretical basis for future therapeutic strategies aimed at modulating MC activity in this condition.

However, our study has several limitations. Previous research has shown that BTK phosphorylation is involved in the activation of signaling pathways such as PLC-γ[306] and STAT3[307]. In our current work, we focused primarily on the protein-level effects of BTK in the context of IC/BPS, and the downstream signaling pathways involved remain to be elucidated in future investigations.


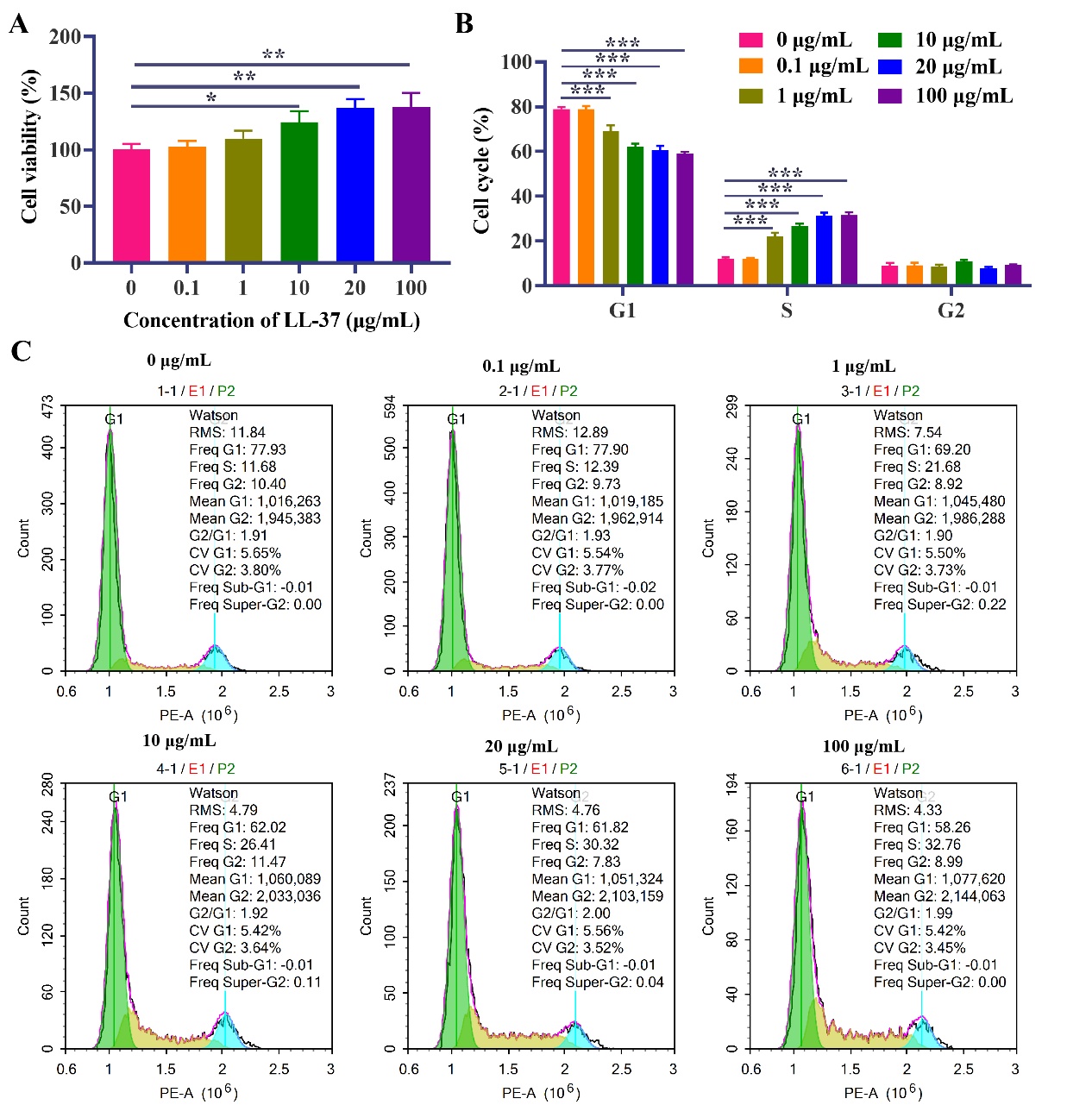


Figure.1 LL-37 promotes the proliferation and cell cycle progression of MCS


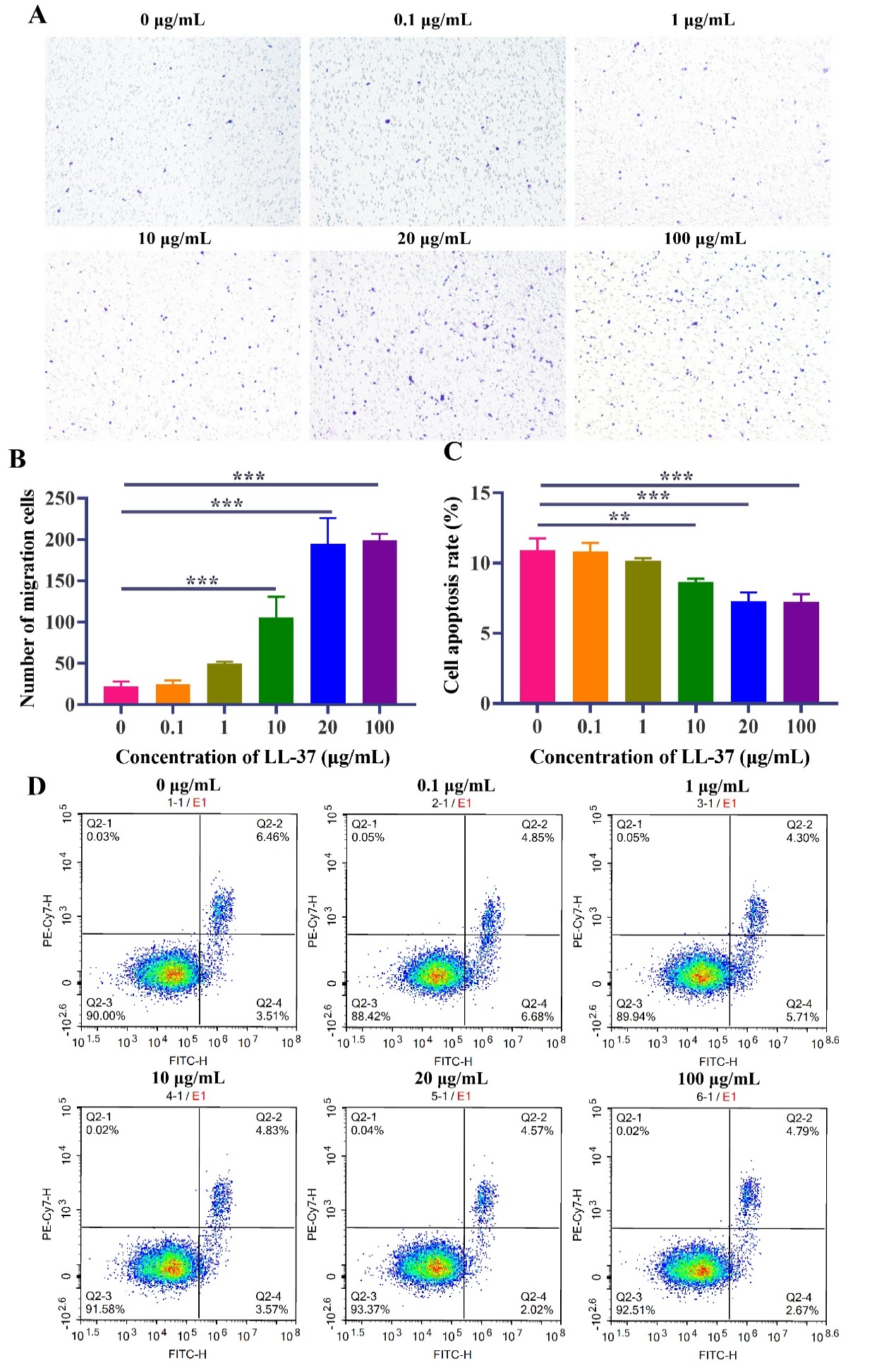


Figure2. LL-37 stimulation promotes MCS invasion and reduces apoptosis

A-B: Transwell chamber was used to detect the invasive ability of MCS. C-D: annexin V-FITC/PI double staining was used to evaluate the apoptosis rate of MCS by flow cytometry** P<0.05，***P<0.05。

1.
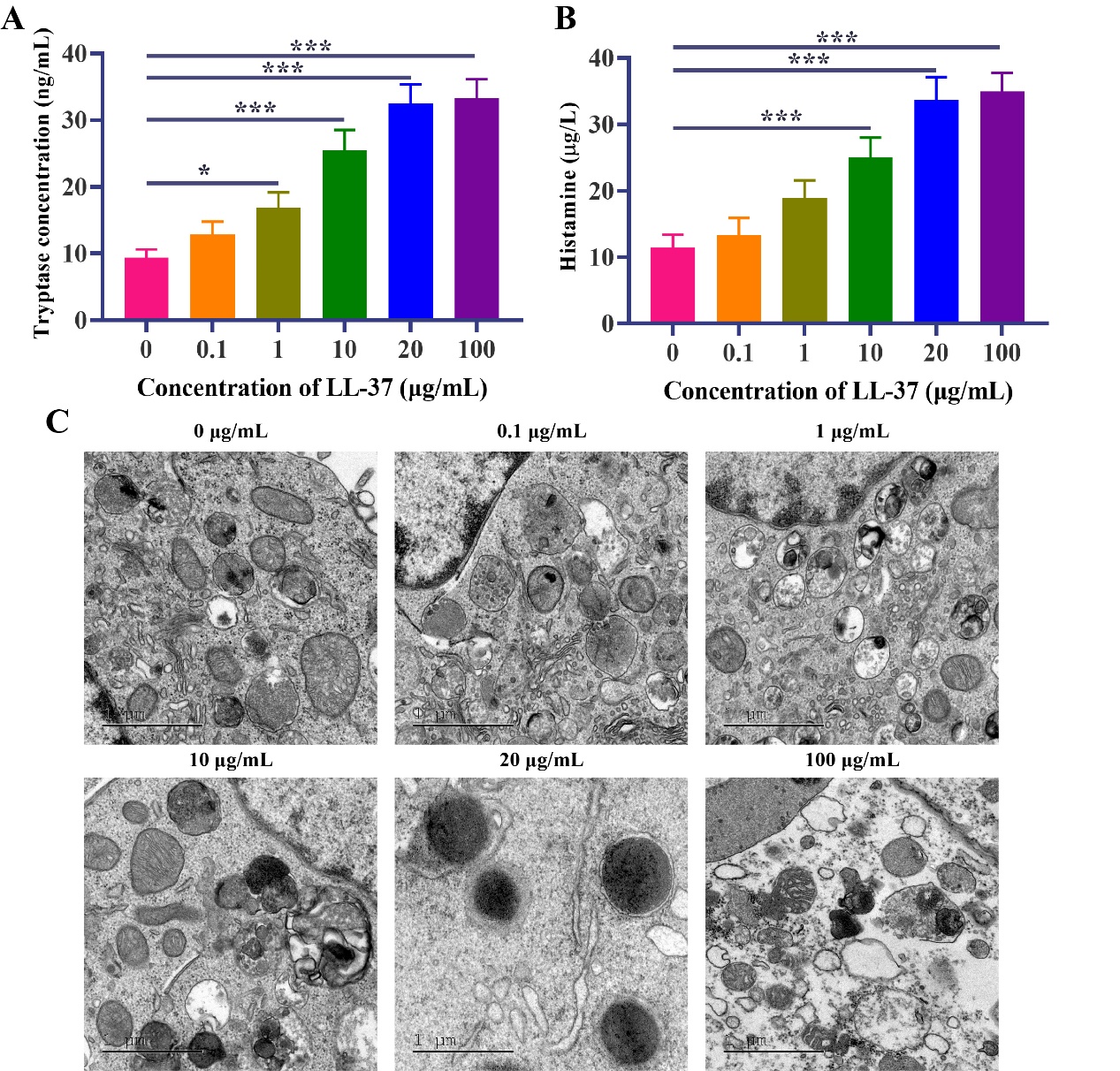


Figure3. LL-37 stimulation promotes degranulation of MCS

A-B: the concentrations of tryptase and histamine in MCs were detected by ELISA. C: The degranulation of cells was observed by TEM. *P<0.05，***P<0.05。
