# Supplementary material for: Role of Bruton’s Tyrosine Kinase in mast cell driven urothelial barrier injury in an LL-37 induced model of interstitial cystitis
Source: Sci Rep. 2026 Apr 30;16:20181. doi: 10.1038/s41598-026-50443-z (PMC13323726; doi:10.1038/s41598-026-50443-z)
Supplement: Supplementary file 2 — Supplementary Material 2 [file 41598_2026_50443_MOESM2_ESM.docx]

**Full-length bolt images**





Figure2. E(C)BTK





Figure2. E(C)Bcl-2





Figure2. E(C)p21





Figure2. E(C)c-Myc





Figure2. E(C)GAPDH





Figure3. A(d)Claudin-1





Figure3. A(d)GAPDH





Figure3. A(d)Occludin





Figure3. A(d)ZO-1





Figure4A(b). BTK


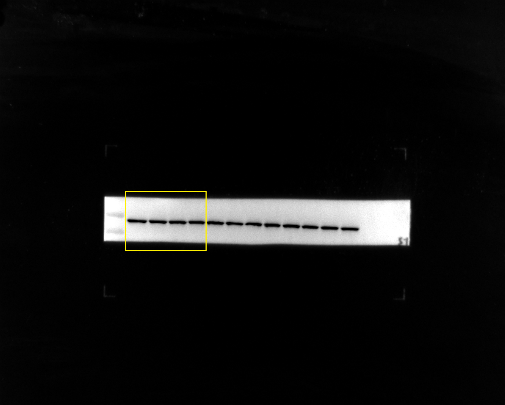


Line(boxed) was used in Figure4A(b).GAPDH





Figure4A(c).BTK





Figure4A(c).GAPDH





Figure4C(d).Claudin-1





Figure4C(d).GAPDH





Figure4C(d).Occludin





Figure4C(d).ZO1
