# Supplementary figures and images for: Role of Bruton’s Tyrosine Kinase in mast cell driven urothelial barrier injury in an LL-37 induced model of interstitial cystitis
Source: Sci Rep. 2026 Apr 30;16:20181. doi: 10.1038/s41598-026-50443-z (PMC13323726; doi:10.1038/s41598-026-50443-z)

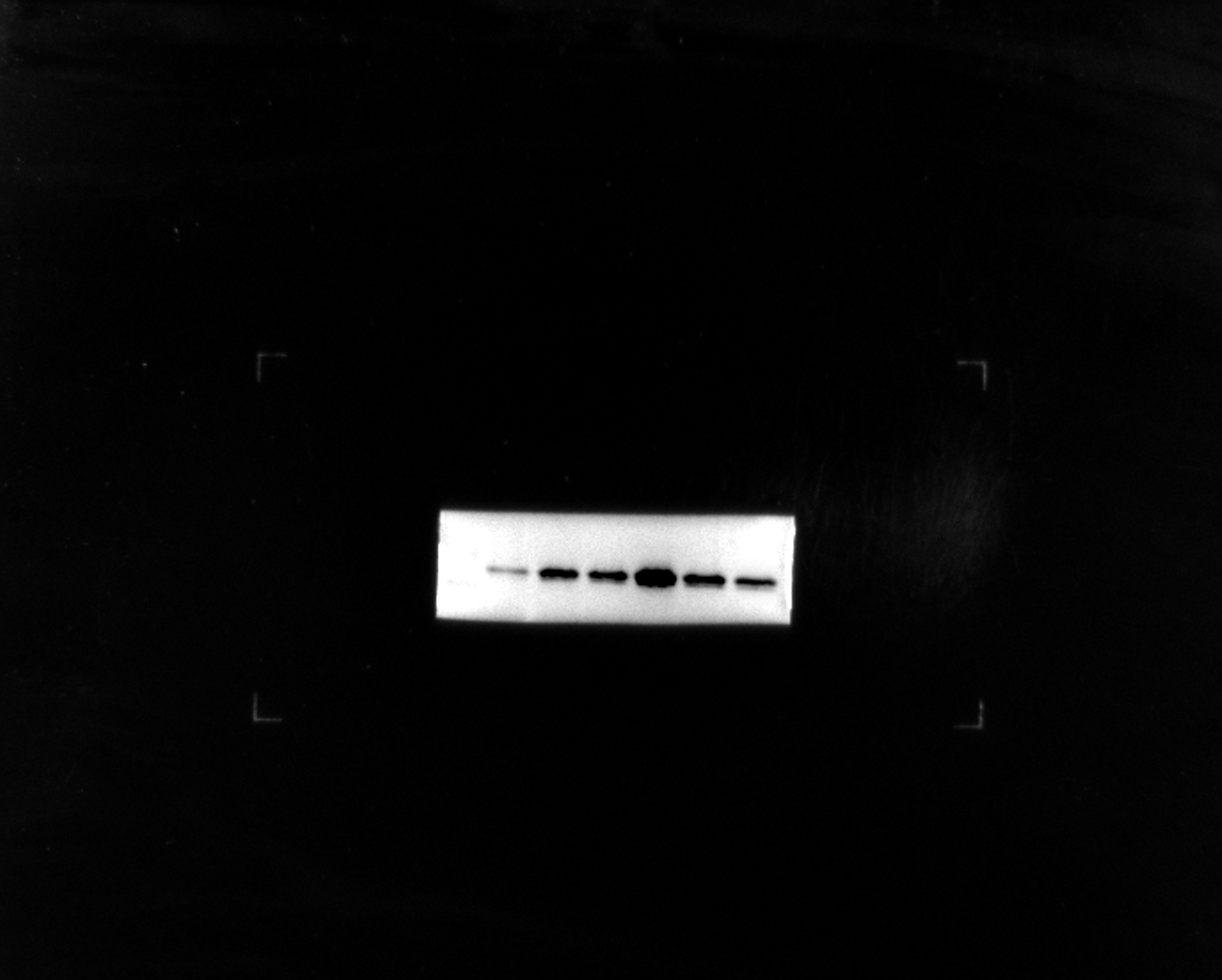

Supplement: Supplementary file 3 — Supplementary Material 3 [file 41598_2026_50443_MOESM3_ESM.tif]

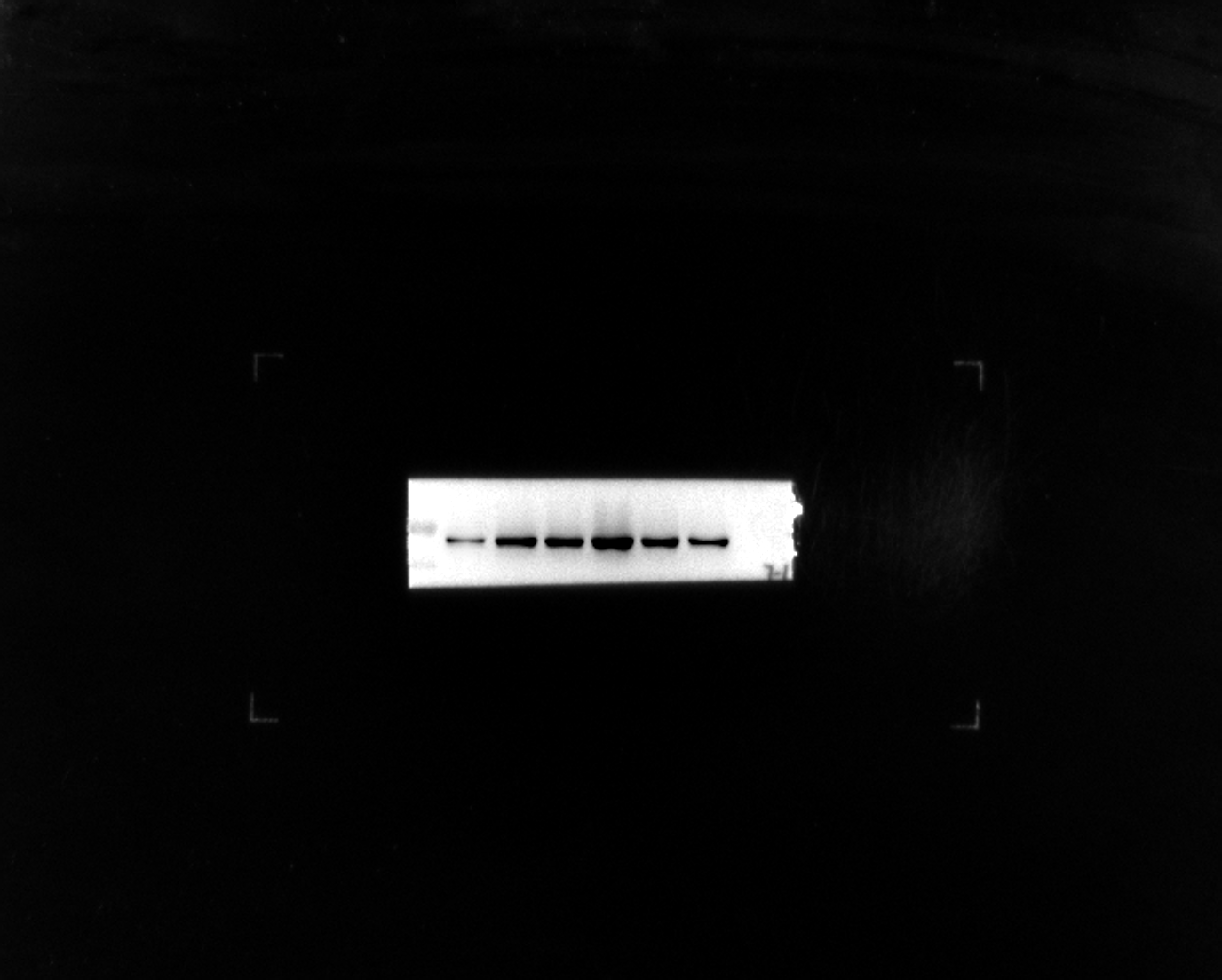

Supplement: Supplementary file 4 — Supplementary Material 4 [file 41598_2026_50443_MOESM4_ESM.tif]

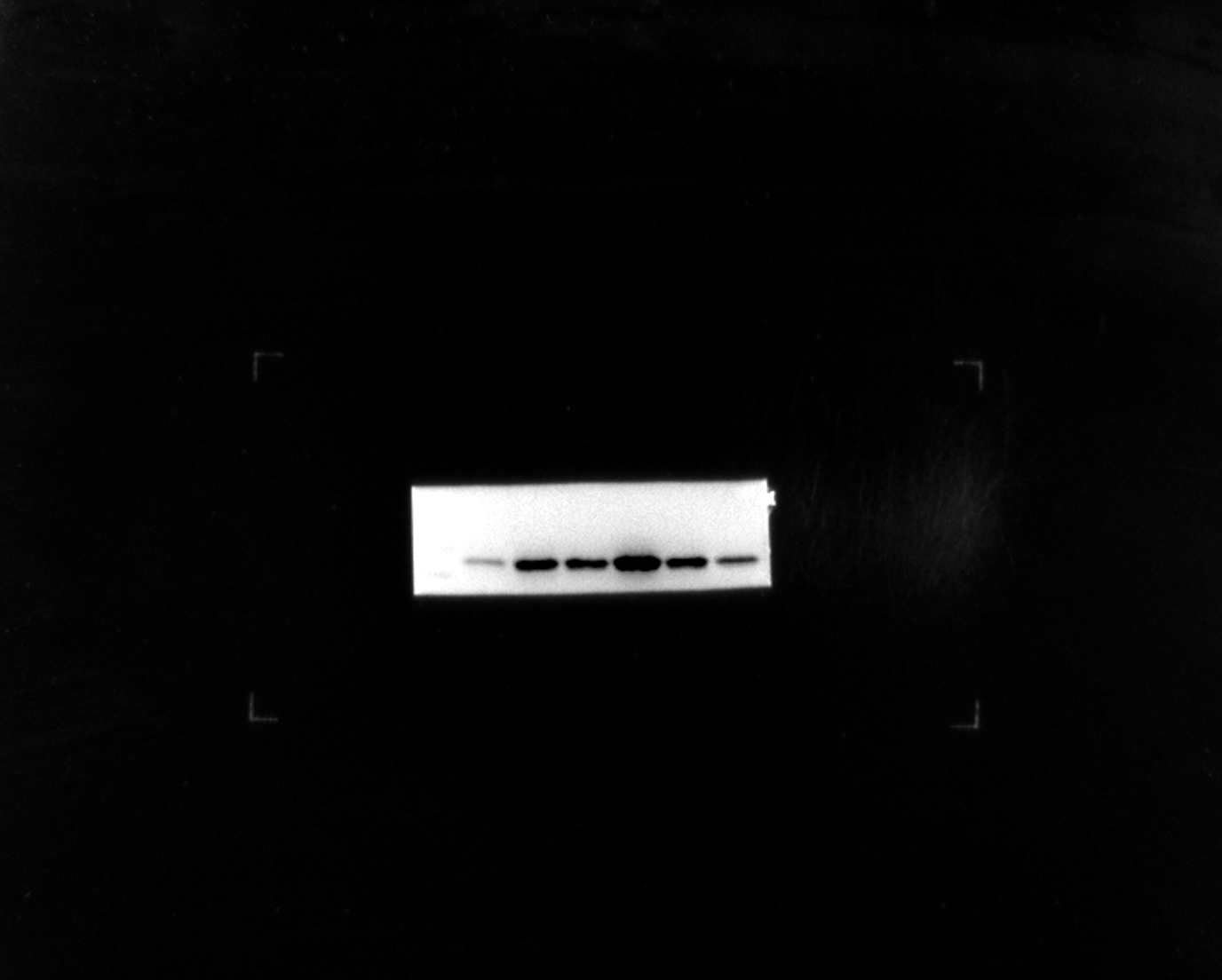

Supplement: Supplementary file 5 — Supplementary Material 5 [file 41598_2026_50443_MOESM5_ESM.tif]

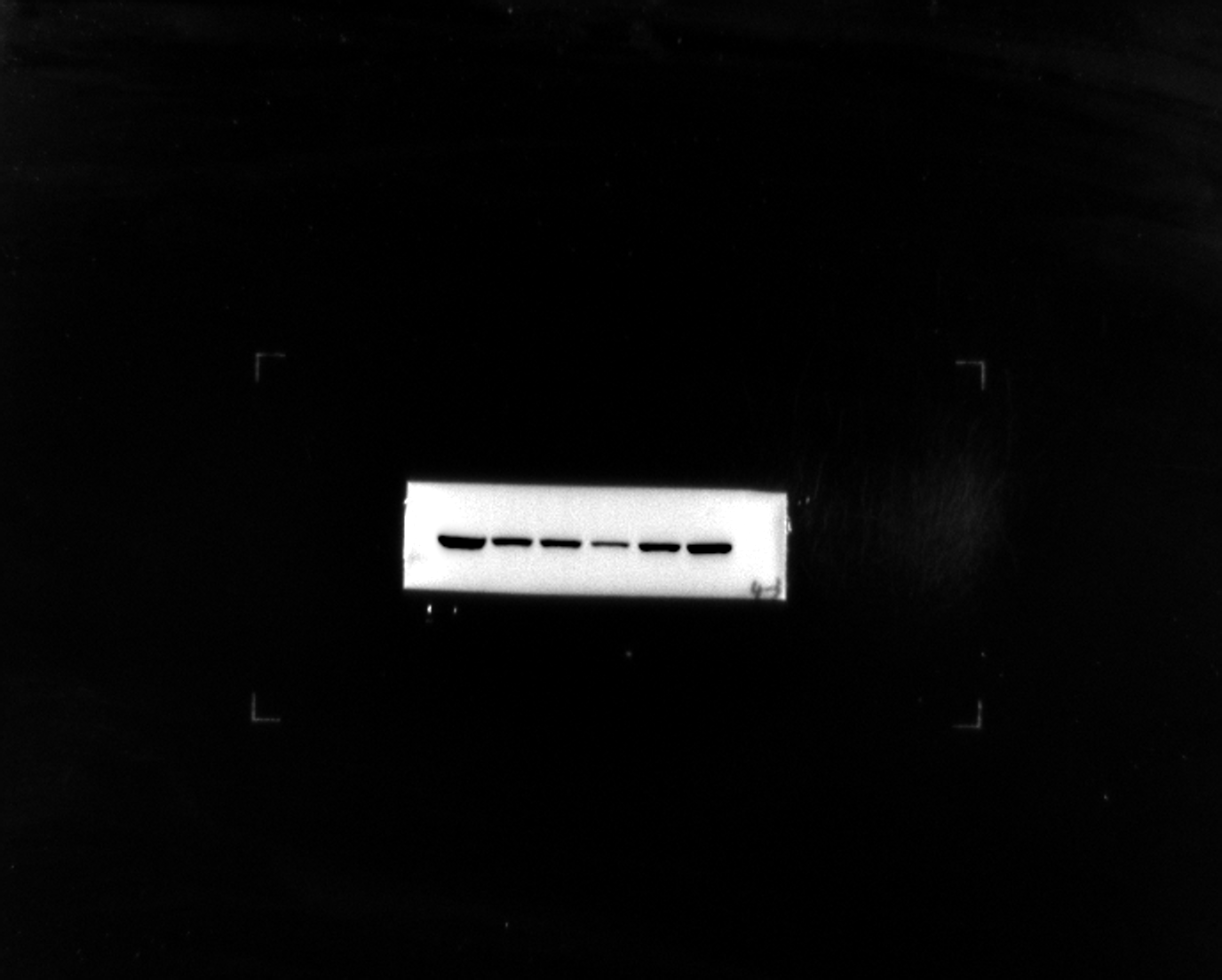

Supplement: Supplementary file 6 — Supplementary Material 6 [file 41598_2026_50443_MOESM6_ESM.tif]

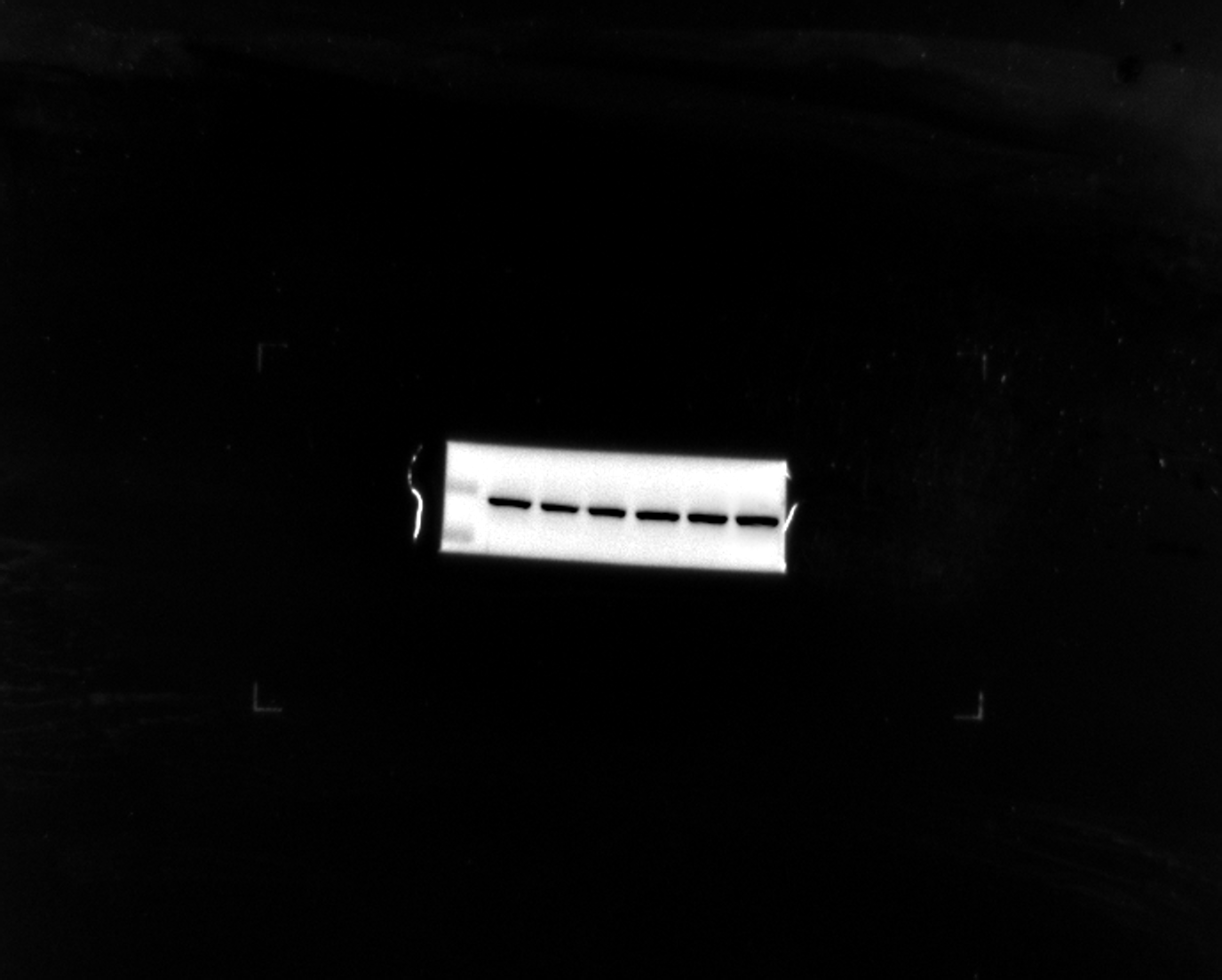

Supplement: Supplementary file 7 — Supplementary Material 7 [file 41598_2026_50443_MOESM7_ESM.tif]

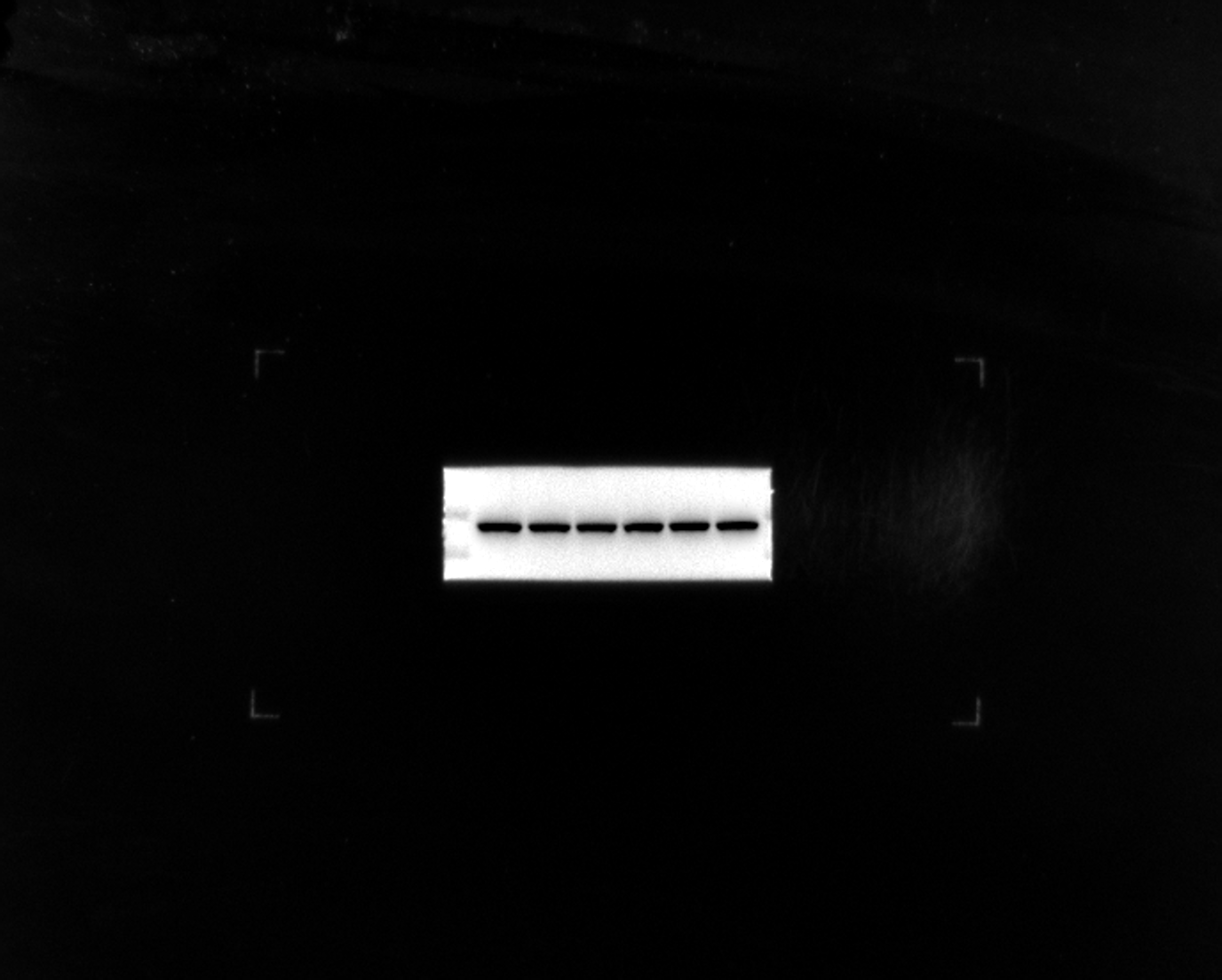

Supplement: Supplementary file 8 — Supplementary Material 8 [file 41598_2026_50443_MOESM8_ESM.tif]

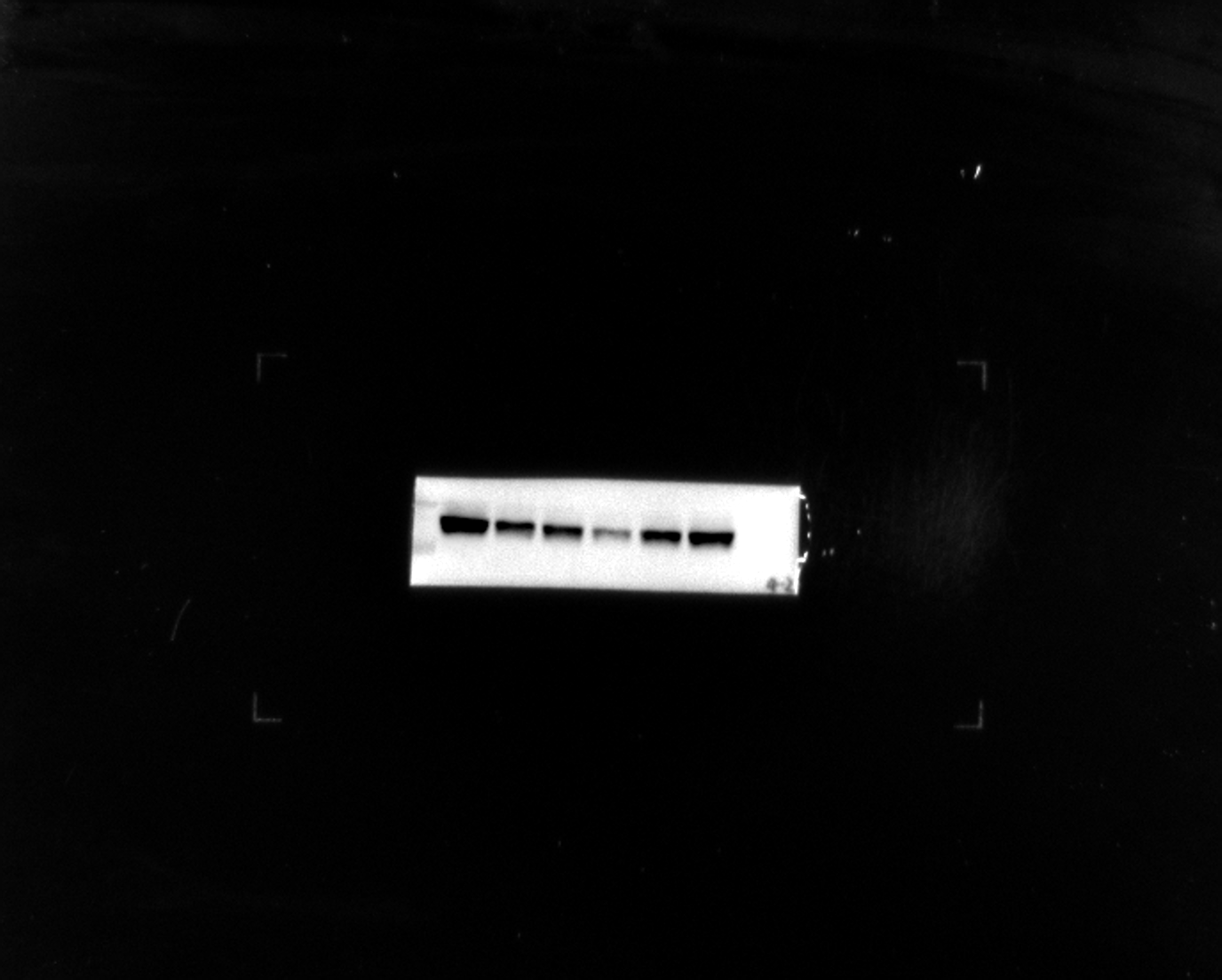

Supplement: Supplementary file 9 — Supplementary Material 9 [file 41598_2026_50443_MOESM9_ESM.tif]

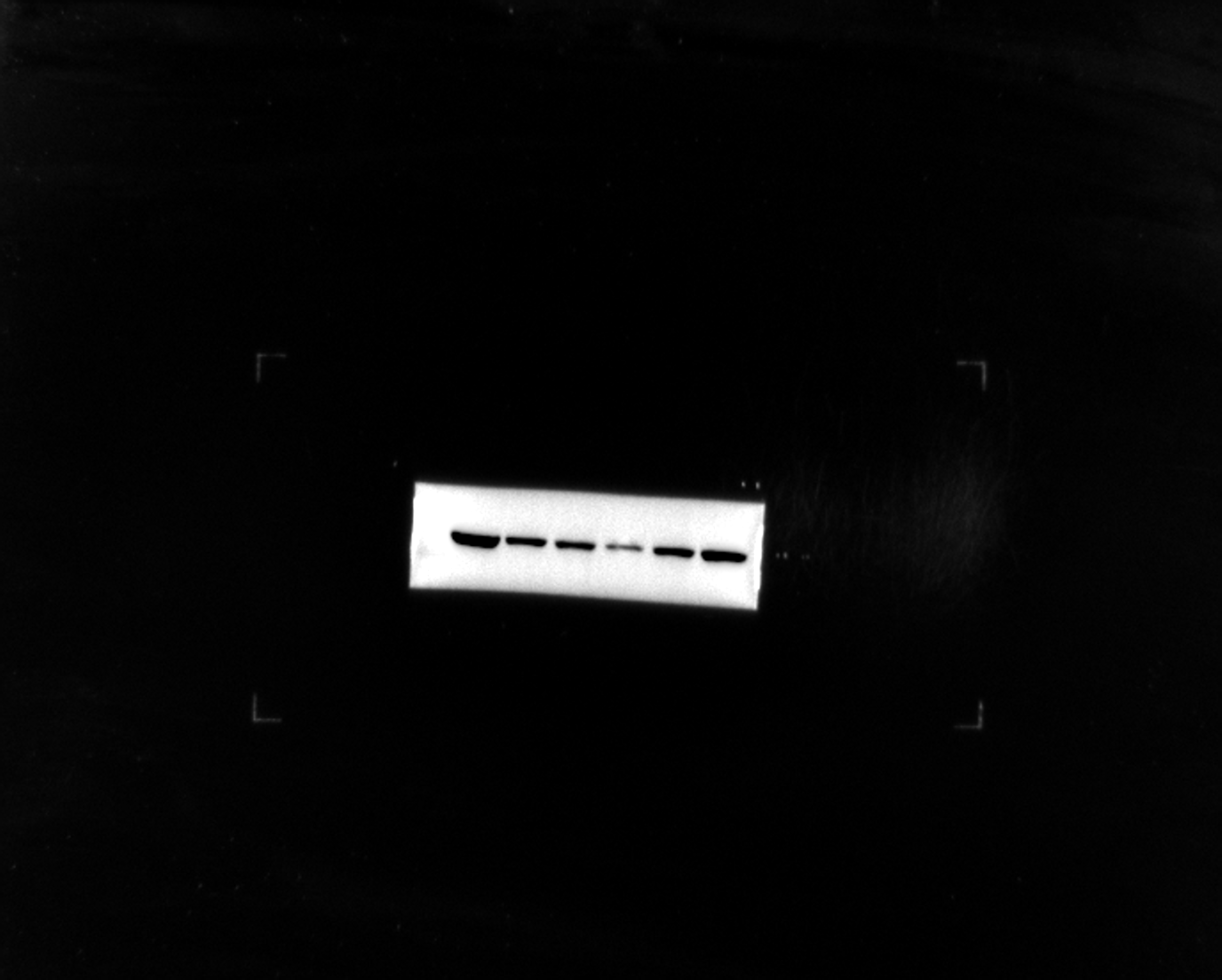

Supplement: Supplementary file 10 — Supplementary Material 10 [file 41598_2026_50443_MOESM10_ESM.tif]

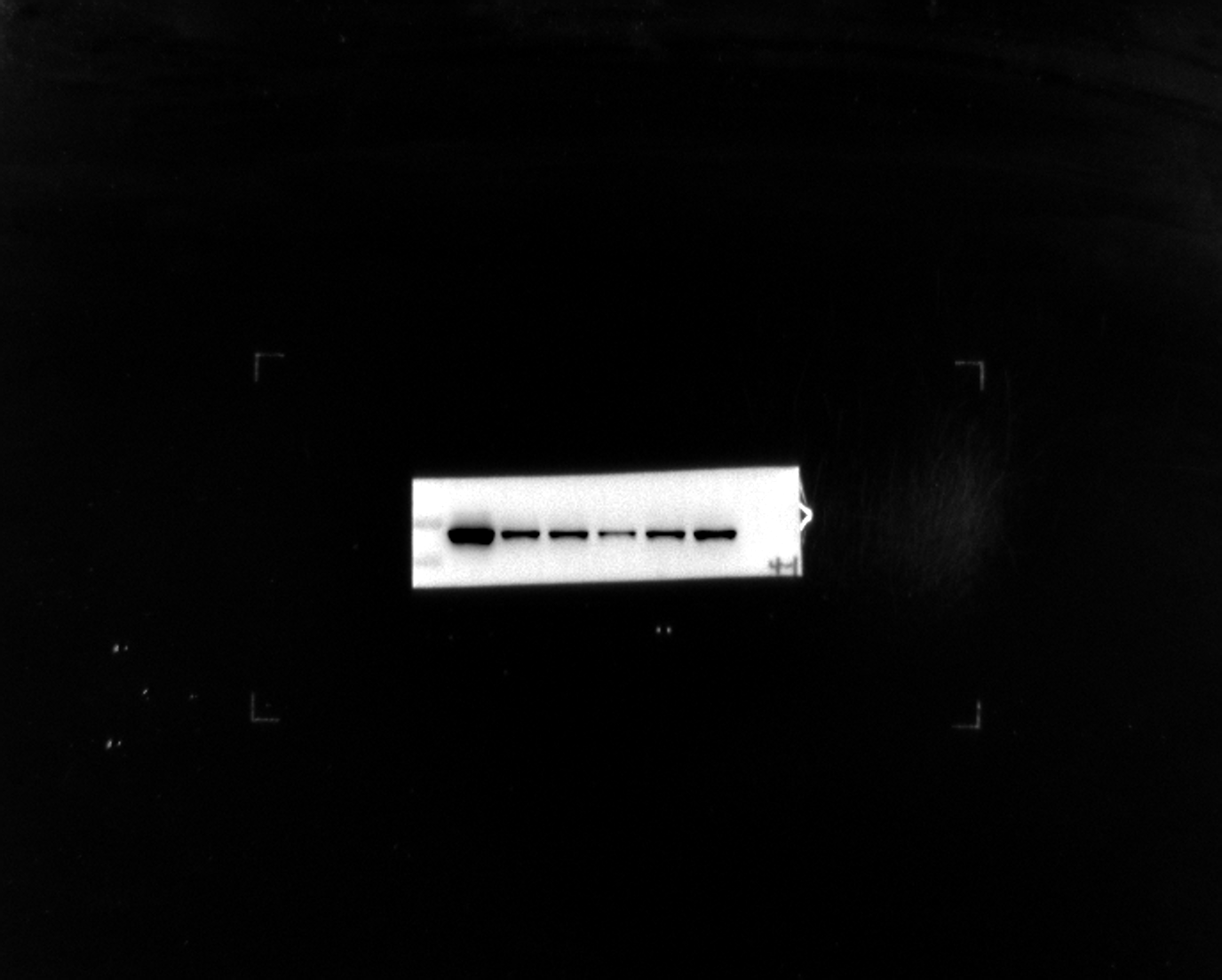

Supplement: Supplementary file 11 — Supplementary Material 11 [file 41598_2026_50443_MOESM11_ESM.tif]

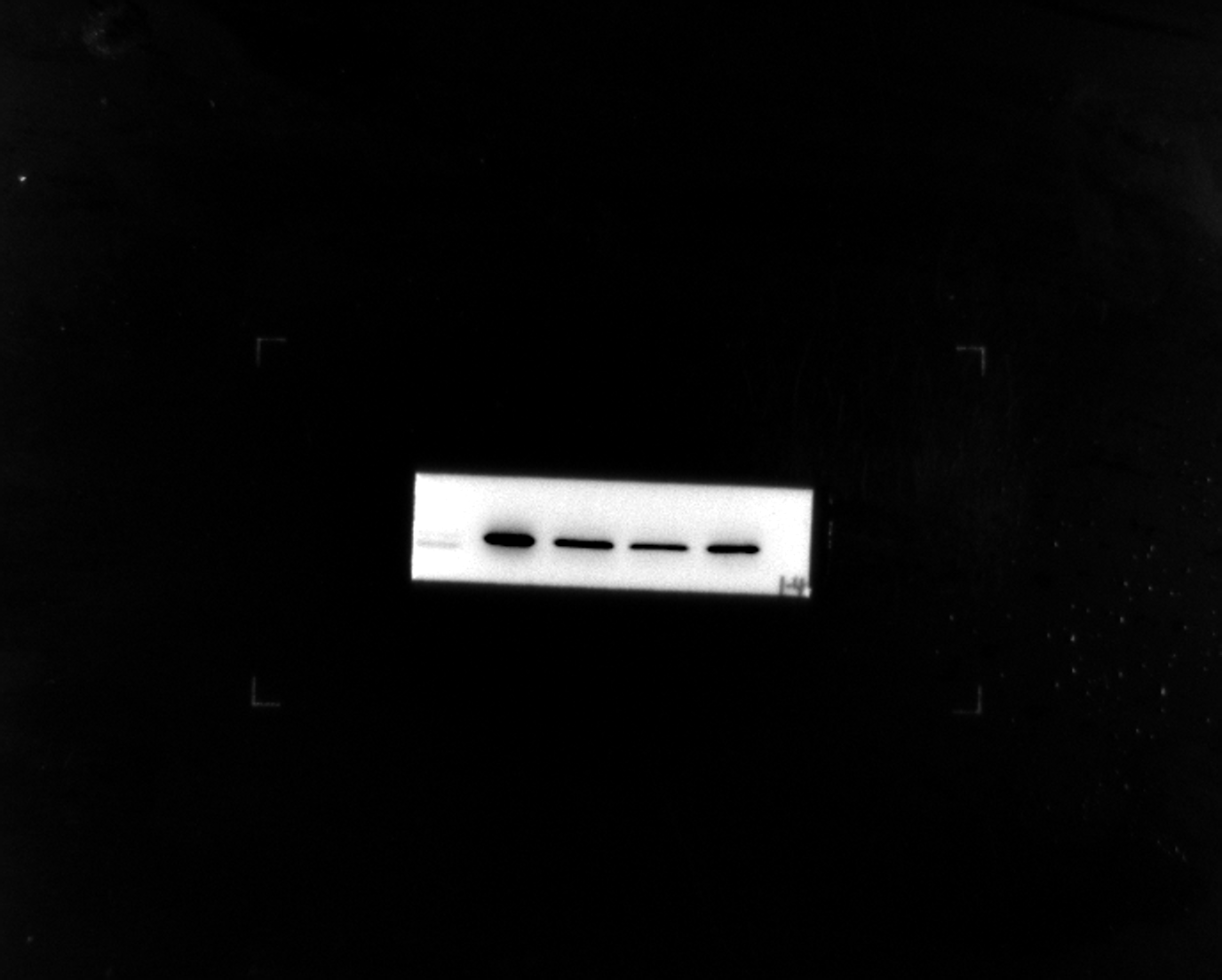

Supplement: Supplementary file 12 — Supplementary Material 12 [file 41598_2026_50443_MOESM12_ESM.tif]

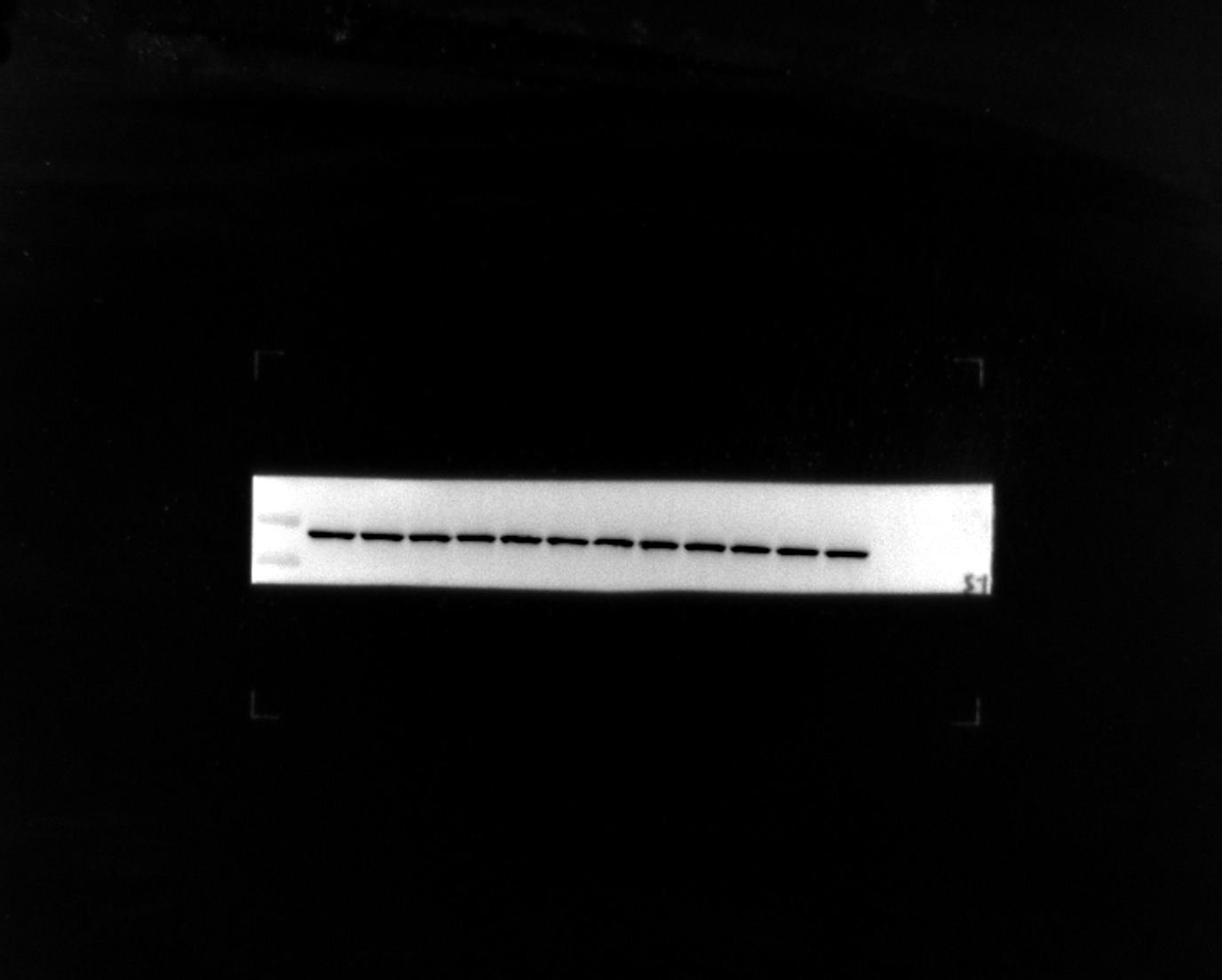

Supplement: Supplementary file 13 — Supplementary Material 13 [file 41598_2026_50443_MOESM13_ESM.tif]

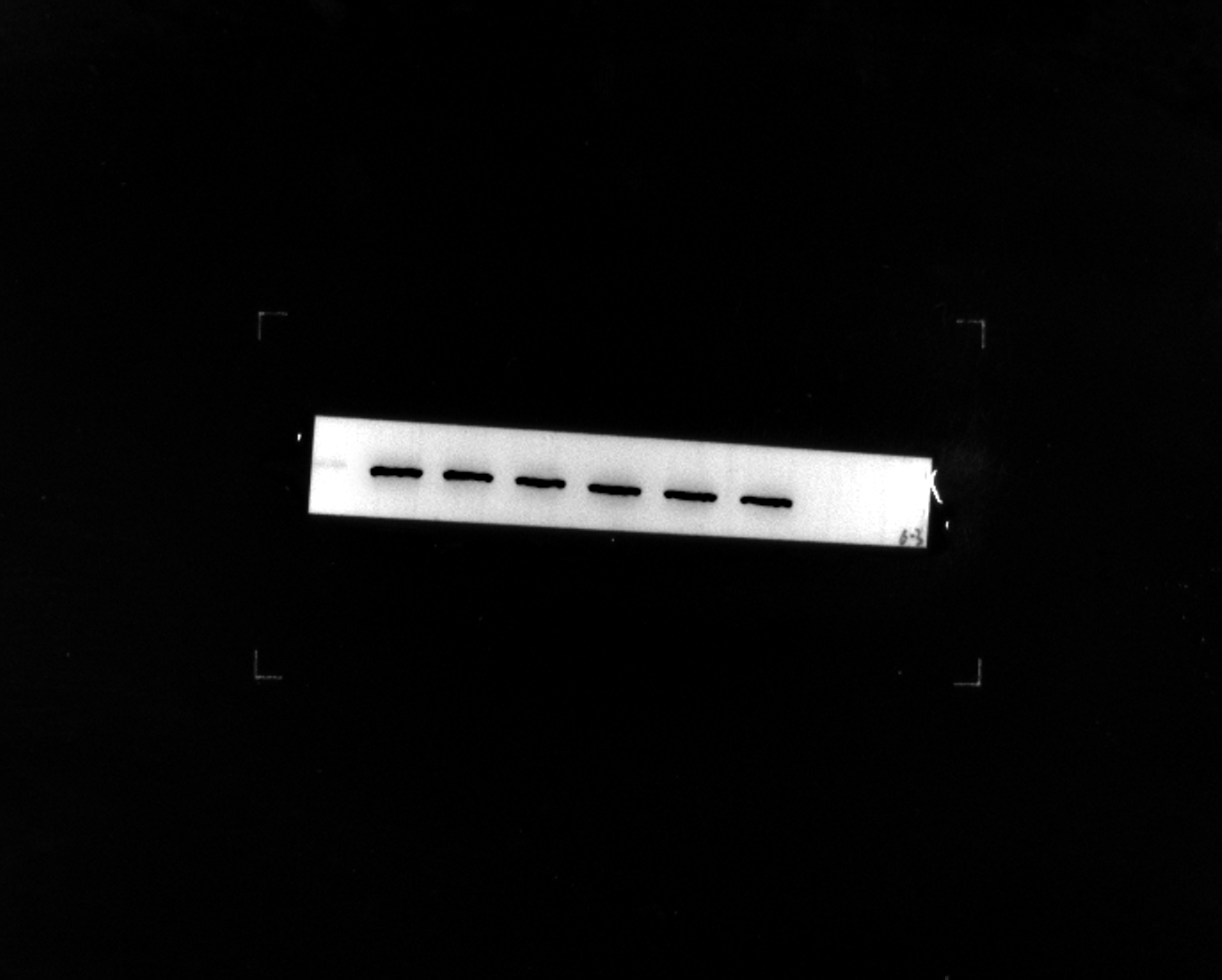

Supplement: Supplementary file 14 — Supplementary Material 14 [file 41598_2026_50443_MOESM14_ESM.tif]

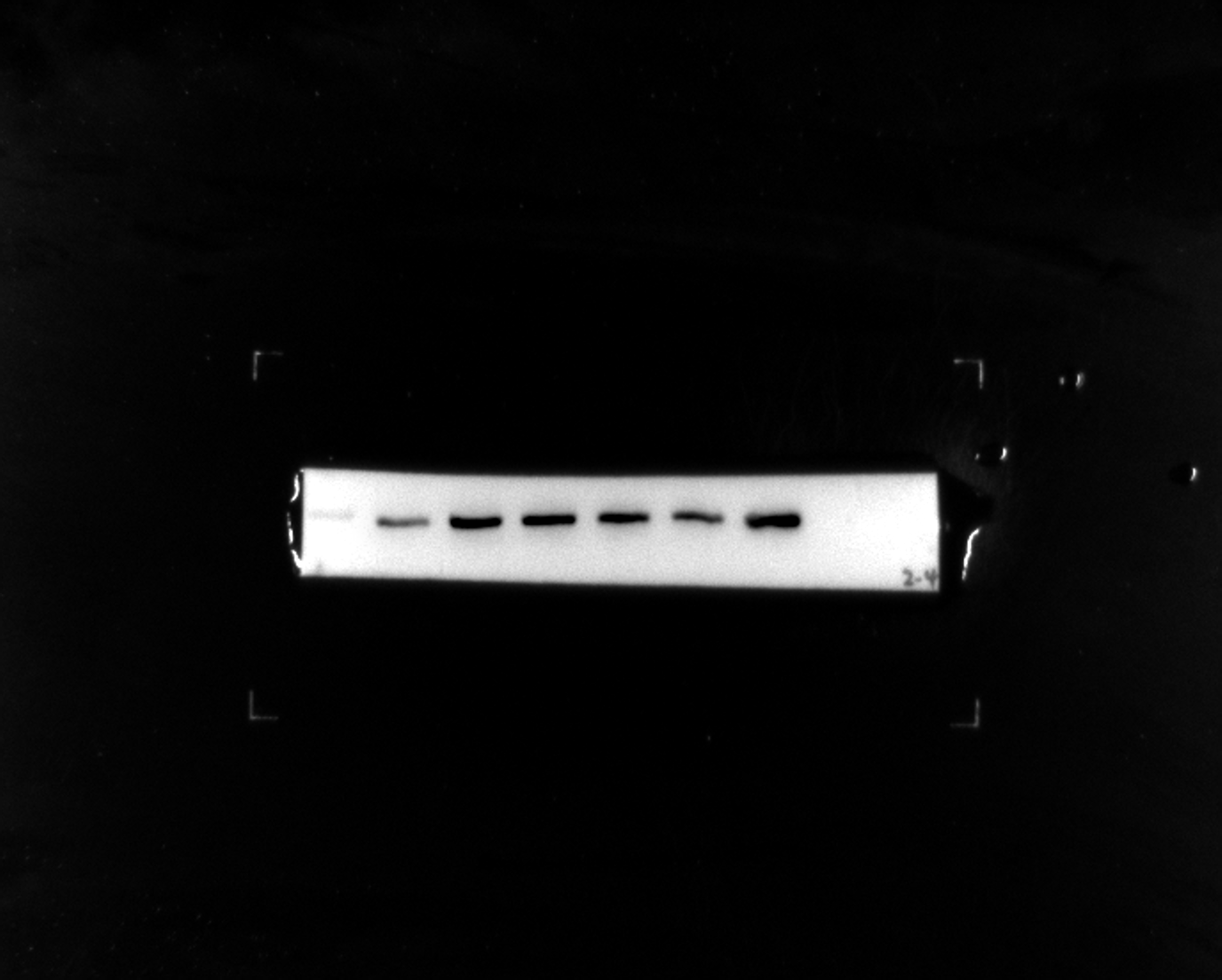

Supplement: Supplementary file 15 — Supplementary Material 15 [file 41598_2026_50443_MOESM15_ESM.tif]

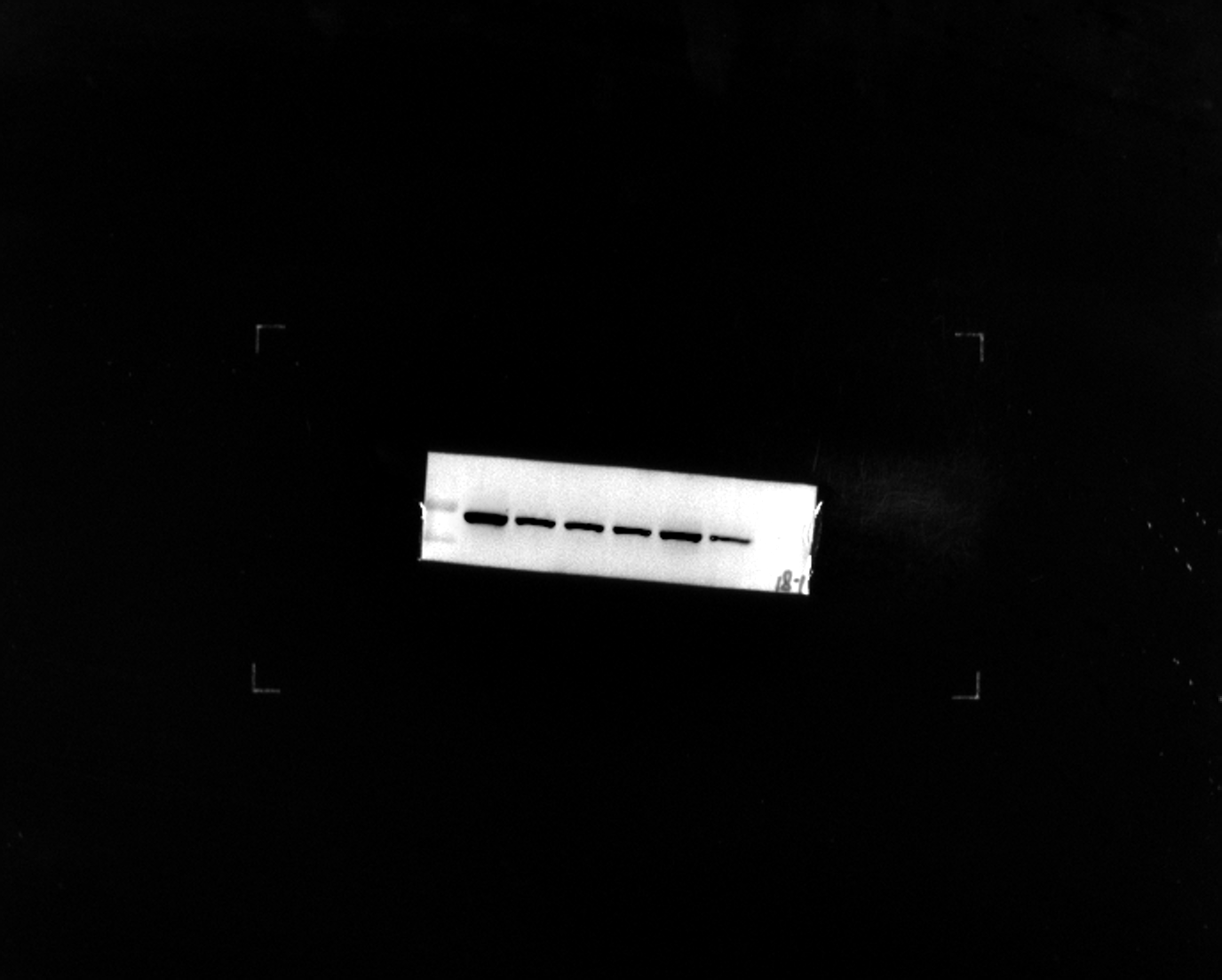

Supplement: Supplementary file 16 — Supplementary Material 16 [file 41598_2026_50443_MOESM16_ESM.tif]

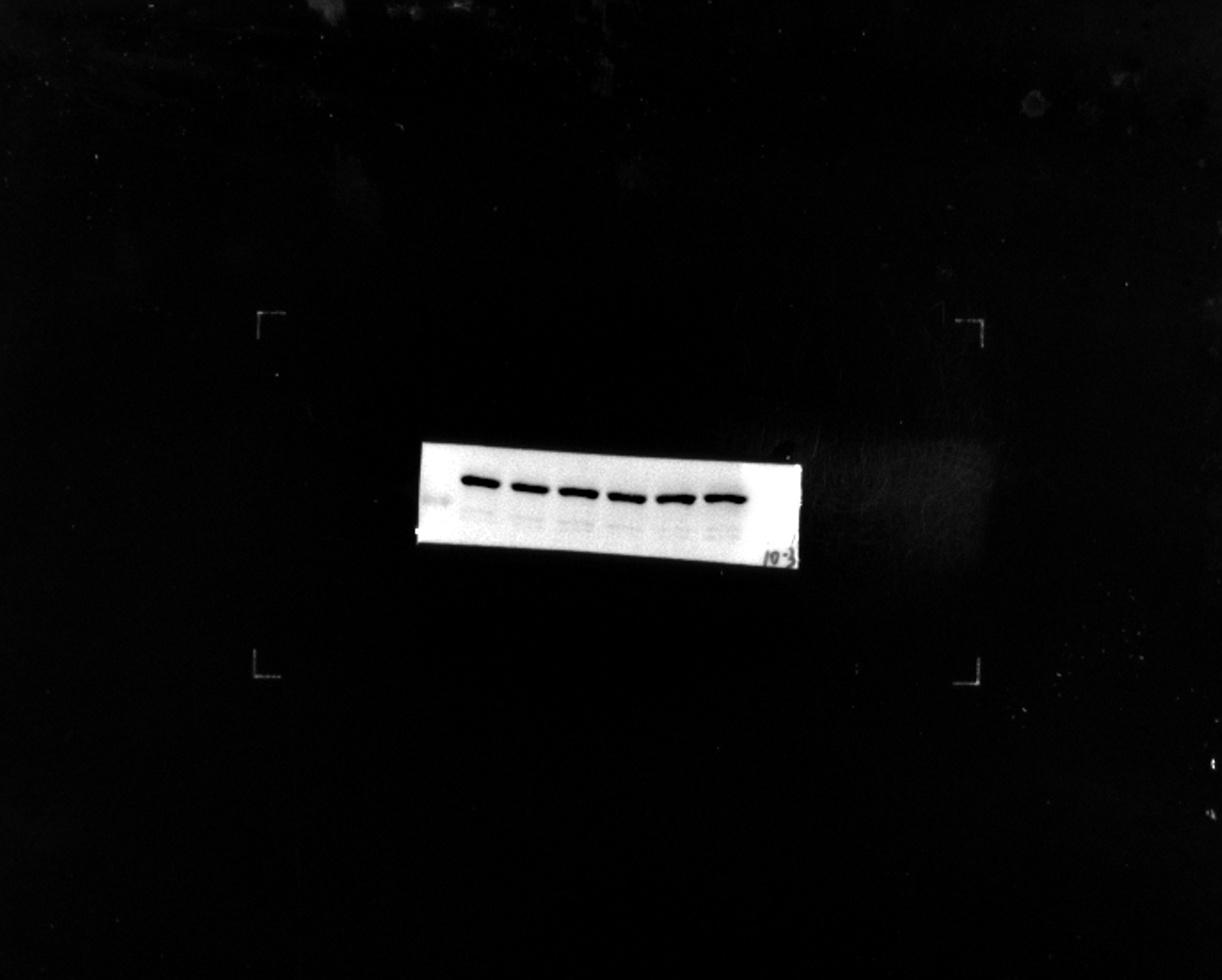

Supplement: Supplementary file 17 — Supplementary Material 17 [file 41598_2026_50443_MOESM17_ESM.tif]

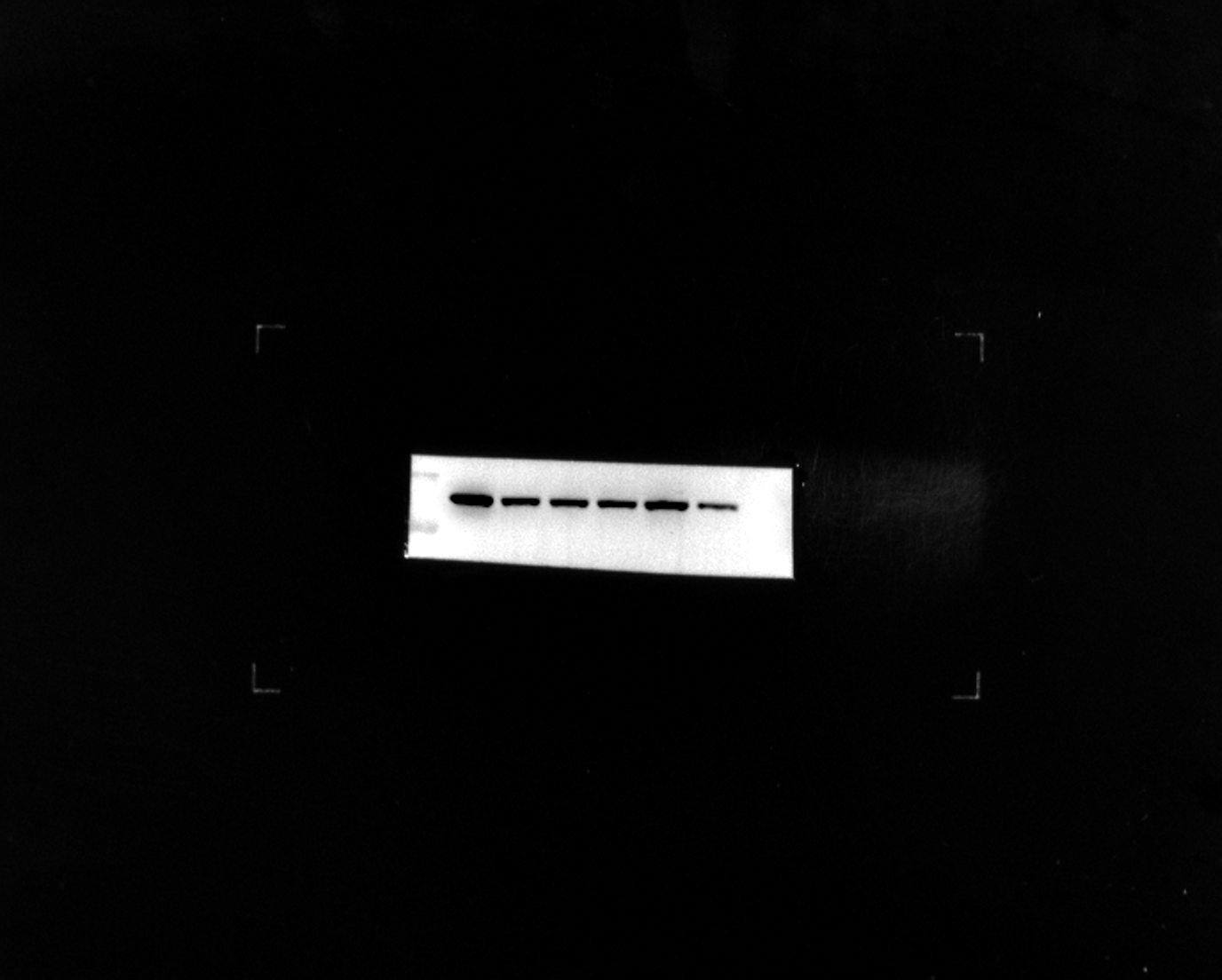

Supplement: Supplementary file 18 — Supplementary Material 18 [file 41598_2026_50443_MOESM18_ESM.tif]

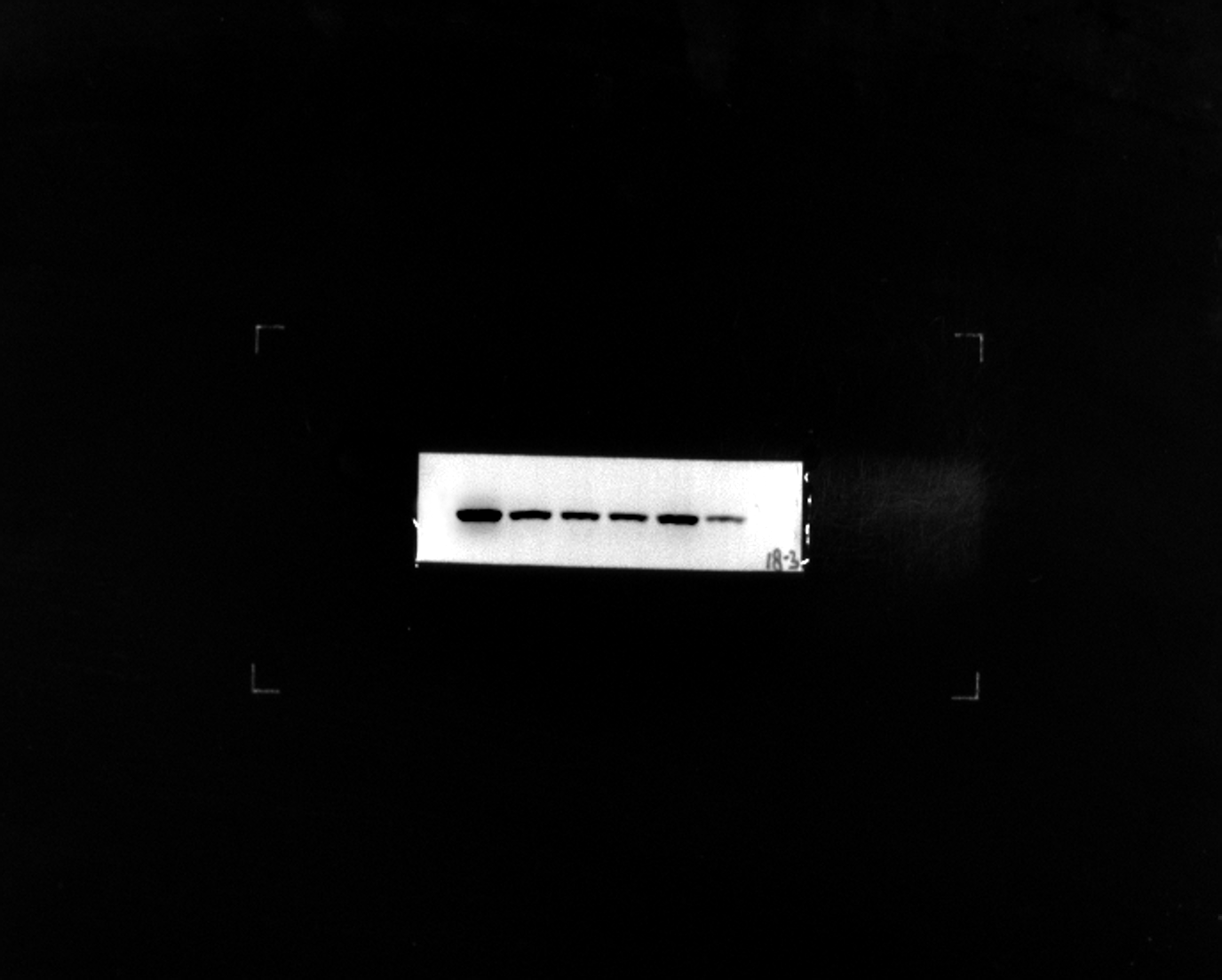

Supplement: Supplementary file 19 — Supplementary Material 19 [file 41598_2026_50443_MOESM19_ESM.tif]
